# Role of hepcidin upregulation and proteolytic cleavage of ferroportin 1 in hepatitis C virus-induced iron accumulation

**DOI:** 10.1371/journal.ppat.1011591

**Published:** 2023-08-16

**Authors:** Kazuyoshi Ohta, Masahiko Ito, Takeshi Chida, Kenji Nakashima, Satoshi Sakai, Yumi Kanegae, Hideya Kawasaki, Takuya Aoshima, Shuji Takabayashi, Hirotaka Takahashi, Kazuhito Kawata, Ikuo Shoji, Tatsuya Sawasaki, Takafumi Suda, Tetsuro Suzuki

**Affiliations:** 1 2nd Department of Internal Medicine, Hamamatsu University School of Medicine, Hamamatsu, Shizuoka, Japan; 2 Department of Microbiology and Immunology, Hamamatsu University School of Medicine, Hamamatsu, Shizuoka, Japan; 3 Department of Regional Medical Care Support, Hamamatsu University School of Medicine, Hamamatsu, Shizuoka, Japan; 4 Department of Molecular Biology, Hamamatsu University School of Medicine, Hamamatsu, Shizuoka, Japan; 5 Core Research Facilities, Research Center for Medical Sciences, The Jikei University School of Medicine, Tokyo, Japan; 6 Institute for NanoSuit Research, Preeminent Medical Photonics Education & Research Center, Hamamatsu University School of Medicine, Hamamatsu, Shizuoka, Japan; 7 Laboratory Animal Facilities & Services, Preeminent Medical Photonics Education & Research Center, Hamamatsu University School of Medicine, Hamamatsu, Shizuoka, Japan; 8 Division of Cell-Free Science, Proteo-Science Center, Ehime University, Matsuyama, Ehime, Japan; 9 Division of Infectious Disease Control, Center for Infectious Diseases, Kobe University Graduate School of Medicine, Kobe, Hyogo, Japan; Heidelberg University, GERMANY

## Abstract

Hepatitis C virus (HCV) is a pathogen characterized not only by its persistent infection leading to the development of cirrhosis and hepatocellular carcinoma (HCC), but also by metabolic disorders such as lipid and iron dysregulation. Elevated iron load is commonly observed in the livers of patients with chronic hepatitis C, and hepatic iron overload is a highly profibrogenic and carcinogenic factor that increases the risk of HCC. However, the underlying mechanisms of elevated iron accumulation in HCV-infected livers remain to be fully elucidated. Here, we observed iron accumulation in cells and liver tissues under HCV infection and in mice expressing viral proteins from recombinant adenoviruses. We established two molecular mechanisms that contribute to increased iron load in cells caused by HCV infection. One is the transcriptional induction of hepcidin, the key hormone for modulating iron homeostasis. The transcription factor cAMP-responsive element-binding protein hepatocyte specific (CREBH), which was activated by HCV infection, not only directly recognizes the hepcidin promoter but also induces bone morphogenetic protein 6 (BMP6) expression, resulting in an activated BMP-SMAD pathway that enhances hepcidin promoter activity. The other is post-translational regulation of the iron-exporting membrane protein ferroportin 1 (FPN1), which is cleaved between residues Cys^284^ and Ala^285^ in the intracytoplasmic loop region of the central portion mediated by HCV NS3-4A serine protease. We propose that host transcriptional activation triggered by endoplasmic reticulum stress and FPN1 cleavage by viral protease work in concert to impair iron efflux, leading to iron accumulation in HCV-infected cells.

## Author summary

Iron plays an important role in many biological processes, including cell proliferation and metabolism, and disruption of iron homeostasis leads to the generation of harmful reactive oxygen species through the Fe2+-triggered Fenton/Haber-Weiss reaction. Iron deposition is often observed in liver tissues of chronic hepatitis C patients, and it has been considered that abnormal iron metabolism is potentially involved in the pathogenesis of HCV. In this study, we show that, in the hepcidin-ferroportin axis, the key mechanism involved in regulation of iron homeostasis, both induction of hepcidin expression and proteolytic cleavage of ferroportin 1 protein induced by HCV infection lead to increased iron levels in the cells, and identify viral factors responsible for the iron accumulation. This is the first study to demonstrate that two mechanisms, transcriptional activation of host cells upon pathogen infection and host protein cleavage by pathogen-derived proteases, work together to result in iron accumulation in host cells. These findings not only deepen our understanding of the mechanisms of HCV-related pathologies, but also lead to the development of new therapies for chronic liver diseases.

## Introduction

Hepatitis C virus (HCV) infection is highly persistent and carries the risk of causing life-long illness including liver cirrhosis and hepatocellular carcinoma (HCC). While HCV can be eliminated in most patients with current antiviral therapies such as direct-acting antiviral (DAA) therapy, there are still an estimated 58 million people worldwide with chronic HCV infection, with about 1.5 million new infections occurring per year [1]. HCV is an enveloped virus with a single-stranded RNA genome of positive polarity that belongs to the *Flaviviridae* family. The precursor polyprotein of HCV, which consists of about 3,010 amino acids, is cleaved into 10 structural and nonstructural (NS) components by cellular signal peptidases and viral proteases, NS2 and NS3-4A, associated with the endoplasmic reticulum (ER) membrane. The NS3-4A serine protease is not only essential for generating mature viral proteins required for HCV replication, but is also considered to suppress the host antiviral immune system by cleaving putative cellular targets [2].

It has been demonstrated that HCV infection induces ER stress triggered by the localization of viral proteins to the ER lumen or membrane and ER-specific modifications in host cells, and the ER stress response is potentially implicated in altered cellular homeostasis, including abnormalities in lipid and iron metabolism. Iron accumulation in liver tissues is observed in patients with chronic hepatitis C [3]. Enhanced liver fibrosis has been reported in HCV-infected patients with stainable iron in liver biopsies compared with controls that have no detectable liver iron [4]. As iron ions move back and forth between trivalent and divalent charges during their transport in the body, they react with oxygen, hydrogen peroxide, and other substances in tissues to create reactive oxygen species (ROS). The combination of free iron and ROS leads to the production of highly toxic hydroxyl radicals via the Fenton/Haber-Weiss reaction, causing severe cellular damage. According to studies of hereditary hemochromatosis, iron overload is highly likely to be a profibrogenic and carcinogenic factor that increases the risk of HCC [5,6]. Nevertheless, the underlying molecular mechanisms of hepatic iron accumulation in HCV-infected livers are still poorly understood.

The iron-transporting membrane protein ferroportin 1 (FPN1), the only known cellular exporter of iron, and hepcidin, a 25-amino acid peptide hormone (hepcidin-25) produced mainly in the liver, play major roles in maintaining iron homeostasis. When hepcidin binds to FPN1, it leads to endocytosis and ubiquitin-dependent degradation of FPN1, controlling the main inflows of iron into plasma. Since FPN1 is the only protein that directly functions in iron export, several studies have shown that FPN1 is less abundant in cancer cells than in non-cancer cells [7–9], suggesting that FPN1 levels may play a role in cancer development. The production of hepcidin in hepatocytes is primarily regulated at the transcriptional level, and is known to be induced by activation of the transcription factor cyclic-AMP responsive element-binding protein hepatocyte specific (CREBH) [10], the BMP-SMAD signaling pathway [11,12], and the IL-6/STAT3 signaling axis [13,14]. There have been a lot of reports on the effects of HCV infection on hepcidin expression, with most studies stating that HCV has a suppressive effect on hepcidin expression [15–18].

In this study, we identified two mechanisms involved in iron accumulation: 1) increased transcription of the hepcidin gene via a transcriptional pathway triggered and activated by ER stress caused by HCV infection, and 2) proteolytic cleavage of FPN1 by viral NS3-4A serine protease. Based on the reported conformational information of FPN1 and the location of the cleavage site, it is possible that cleavage by NS3-4A impairs the iron-exporter function of FPN1.

## Results

### Elevated levels of iron ion and hepcidin expression in HCV-infected cells

**Fig 1:**
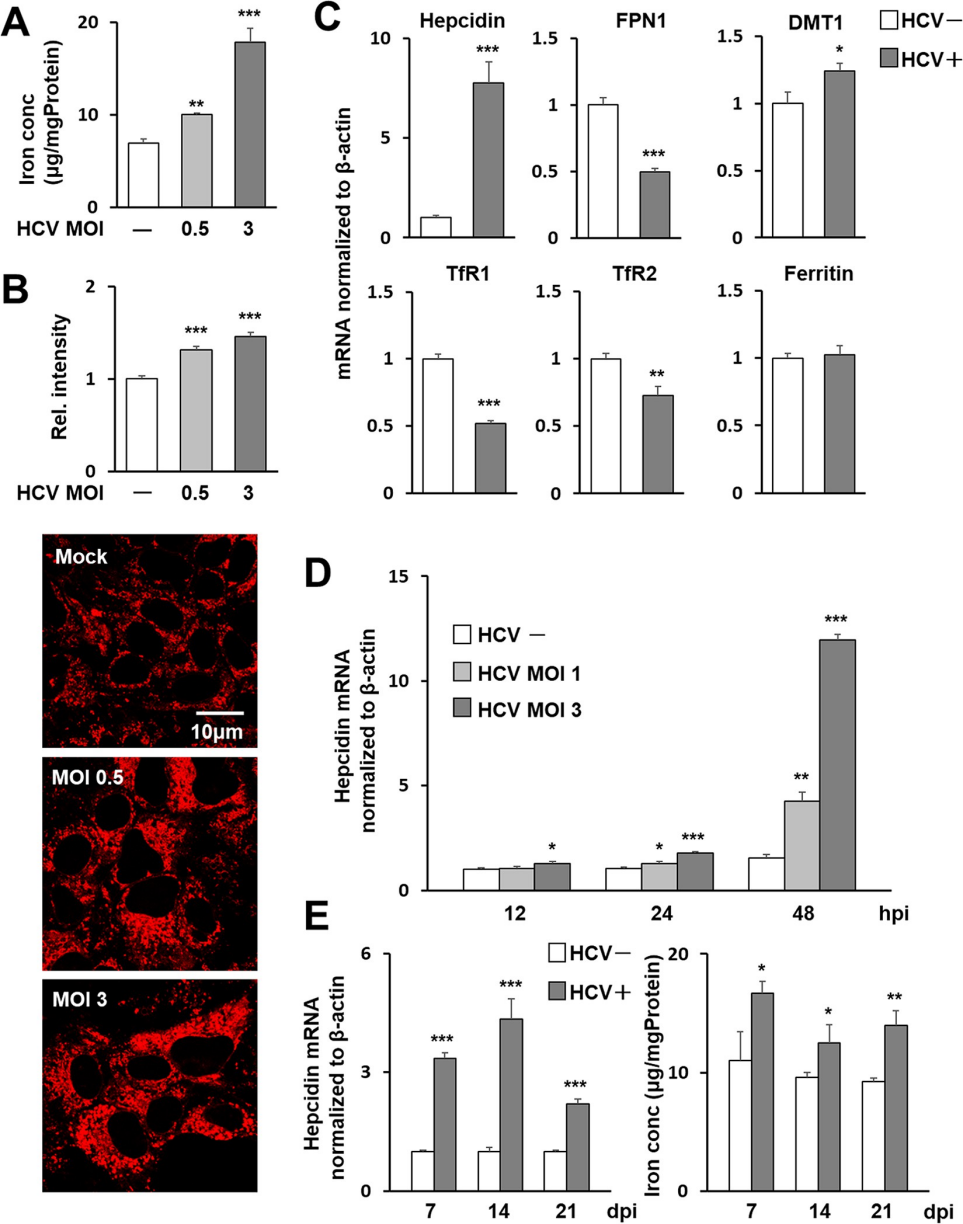
Induction of hepcidin gene expression and intracellular iron accumulation by HCV infection. Iron concentrations in HCV-infected Huh7.5.1 cells 3 days post-infection (dpi) at MOIs of 0, 0.5, and 3 were evaluated through (A) measurement of Fe^2+^ and Fe^3+^ in cell lysates using the Nitroso-PSAP method and (B) measurement of relative intensity corresponding to Fe^2+^ using the fluorescent probe FeRhoNox-1. (B) For each group, cytoplasmic luminescence intensities of cells stained with the fluorescence probe were measured in Huh7.5.1 cells and the relative intensities were graphed with the average intensity of the uninfected-cell group as 1. Representative images of the cells used to measure luminescence intensities are shown at the bottom of the graph. (C) mRNA expressions of hepcidin, FPN1, DMT1, TfR1, TfR2 and ferritin in infected cells 3 dpi (MOI = 0.5, HCV+) and cells without infection (HCV-) were analyzed using qRT-PCR. (D) Time-course changes in hepcidin gene expression induced by HCV infection at MOIs of 1 and 3 were determined. At 12, 24, and 48 hpi, hepcidin mRNA was quantified as above. (E) In long-term cultures of infected cells, hepcidin mRNA was measured at 7, 14, and 21 dpi at MOI of 0.5. Values obtained from mock-infections at each point were set as 1. Intracellular iron concentrations were determined by the Nitroso-PSAP. (A)-(E) Results represent the means with SD from three independent measurements. Student’s *t* test; *P<0.05, **P<0.01, ***P <0.001.

To determine how HCV infection affects iron levels in host cells, the Nitroso-PSAP method (Fig 1A) and FeRhoNox-1 fluorescence intensity (Fig 1B) were used to quantitatively determine the concentrations of free ferrous and ferric iron in HCV-infected cells. An increase in intracellular iron concentration was observed after virus infection in a titer-dependent manner. Increased iron concentrations due to HCV infection were also observed by histological detection specific to ferrous iron with a turn-on fluorescent probe (Fig 1B). When the gene expression of iron regulation-related factors such as hepcidin, ferroportin 1 (FPN1), divalent metal transporter 1 (DMT1), transferrin receptor 1 (TfR1) and TfR2 and ferritin were compared in HCV-infected and -uninfected cells, the increase in expression of the hepcidin gene due to infection was most pronounced, with expression increasing up to 5-fold or more in infected cells compared to uninfected cells (Fig 1C). The expression level of ferritin was unchanged or increased only slightly by HCV infection (S1 Fig). This appears to be consistent with around 1.4-fold change in intracellular iron content in HCV infection (Fig 1A and 1B). The induction of hepcidin gene expression by HCV infection was observed at 24 h post-infection (hpi), with a marked increase in its expression proportional to the amount of viral infection at 48 hpi (Fig 1D). As a control experiment, we inoculated a concentrate of the culture supernatant of uninfected cells and confirmed that it did not affect hepcidin expression as well as the mock control (S2 Fig). Analysis of HCV-infected long-term culture systems in which Huh7.5.1 cells can continue to be cultured without passaging for several weeks in the presence of DMSO [19,20] also showed increased expression of the hepcidin gene and intracellular iron accumulation in cells at 7, 14, and 21 days of HCV infection (Fig 1E).

### Critical role of CREBH in the transcriptional upregulation of BMP6 and hepcidin caused by HCV infection

**Fig 2:**
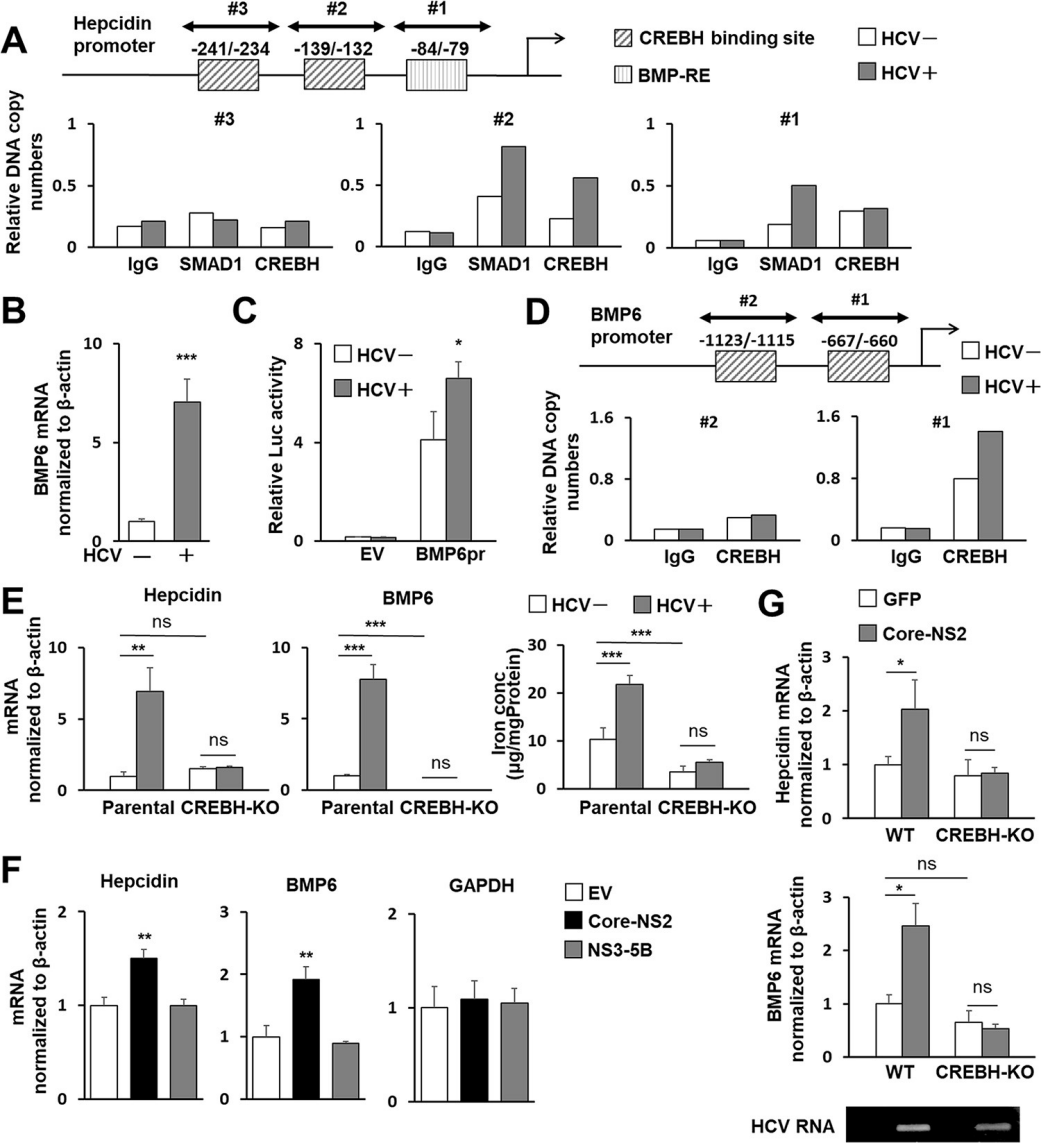
Role of CREBH in hepcidin and BMP6 expression induced by HCV infection or protein expression. (A) Nuclear fractions from infected cells 3 dpi (MOI = 0.5) and cells without infection were used for ChIP assays. Protein-DNA complexes were immunoprecipitated with antibodies against either SMAD1 or CREBH or with rabbit IgG, analyzed by qPCR using three primer sets: Hepcidin pr ChIP set #1-#3. The sites potentially recognized by CREBH or BMP6 are indicated. (B) BMP6 mRNA in cells with and without HCV infection was analyzed using qRT-PCR. (C) BMP6 promoter reporter (BMP6pr) or basal reporter (EV; empty vector) was transfected into cells with HCV infection at 1 dpi and without infection. Firefly luciferase activities normalized by *Renilla* luciferase in cells at 2 days post-transfection (dpt) were determined. (D) ChIP assays and immunoprecipitations were performed as described in (A) with anti-CREBH antibody against or rabbit IgG. Precipitates were analyzed for qPCR using two primer sets: BMP6 pr ChIP set #1 and #2. The CREBH-binding sites are indicated. (E) CREBH-KO and parental Huh7.5.1 cells were cultured with or without HCV infection for 3 days, after which the mRNA of hepcidin (left) and BMP6 (middle) were quantified and the iron concentration (right) was measured by the Nitroso-PSAP. (F) Cells were transfected with a plasmid expressing Core-NS2 or NS3-5B or an EV. At 2 dpt, mRNAs of hepcidin, BMP6, and GAPDH were quantified. (G) Eight-week-old male WT and Creb3l3^−/−^ mice were intravenously injected with AdV expressing Cre recombinase (AdEFNCre) together with AdV expressing either GFP (AdEFLNLGFP) or Core-NS2 (AdEFLNLHCVCore/NS2). At 5 dpi, total RNAs from liver tissues were extracted, followed by determining hepcidin and BMP6 mRNAs. Expression of HCV RNA was detected by RT-PCR. (B, E-G) Results represent the means with SD from three independent measurements. Student’s *t* test; *P<0.05, **P<0.01, ***P <0.001.

It has been reported that transcription factors such as SMAD1 and CREBH bind to the hepcidin promoter sequence, thereby positively regulating its gene expression [10–12]. We analyzed the effect of HCV infection on the recruitment of transcription factors to the hepcidin promoter in cells by chromatin immunoprecipitation (ChIP). The amplified DNA fragments containing the CREBH binding site region in nt -139/-132 (TGACACAA) and BMP-RE region in nt -84/-79 (GGCGCC) were detected after immunoprecipitation with anti-CREBH and anti-SMAD1 antibodies, respectively. These DNA levels were clearly higher in HCV-infected cells than in uninfected cells (Fig 2A).

Bone morphogenetic proteins (BMPs) are members of the TGFβ superfamily, and their binding to BMP type I receptor triggers activation of the downstream Smad1/5/8 signaling cascade, leading to transcription of target genes [21]. The BMP6-SMAD signaling pathway is a central regulator of hepcidin expression. We observed marked increases in hepcidin mRNA expression 6 h after the addition of recombinant BMP6 to Huh7.5.1 cells (S3A Fig) and in BMP6 mRNA expression in HCV-infected cells (Fig 2B). Enhancement of BMP6 promoter activity by HCV infection was also found using a luciferase reporter assay (Fig 2C). Introduction of a BMP6-expressing plasmid into the cells increased hepcidin mRNA expression (S3B Fig), while introduction of BMP6 siRNA decreased hepcidin mRNA expression (S3C Fig). These results indicate that BMP6 expressed in hepatocyte-derived cells is potentially involved in the regulation of hepcidin expression.

Transcription factor binding site prediction revealed that there are two nucleotide sequences mimicking the CRE consensus site on the BMP6 promoter at -1,123/-1,115 (CCACGTGT) and -667/-660 (CCACGTTT). ChIP assay was used to analyze whether CREBH binds to these consensus-like sites, and if so, whether HCV infection affects CREBH-BMP6 promoter binding. Quantitative PCR using CREBH antibody precipitates as templates showed that among the two CRE-like sequences, the -667/-660 site within the BMP6 promoter is likely to interact with CREBH, and that this CREBH-BMP6 promoter interaction is enhanced by HCV infection (Fig 2D).

The involvement of CREBH in the increased expression of BMP6 and hepcidin by HCV infection was further analyzed using CREBH-knockout (CREBH-KO) cells constructed by the CRISPR-Cas9 system [22]. While CREBH-KO Huh7.5.1 cells showed susceptibility to HCV infection comparable to the parental non-knockout cells, no upregulation of BMP6 or hepcidin mRNA expression by HCV infection was observed (Fig 2E), suggesting that CREBH plays a pivotal role in the regulation of gene expression of BMP6 and hepcidin, especially in their upregulation induced by HCV infection. Coupled with the complete cancellation of the induction of hepcidin expression, the increase in intracellular iron levels associated with HCV infection was also considerably suppressed in CREBH-KO cells. Nevertheless, a moderate increase in iron levels was observed in HCV-infected knockout cells (Fig 2E), suggesting that there may be a hepcidin-independent mechanism for intracellular iron accumulation caused by HCV infection.

In our previous study, we found that CREBH is activated by the expression of the Core-NS2 polyprotein among HCV proteins [23]. As expected, transfection of human hepatoma cells with the Core-NS2 expression plasmid enhanced the expression of BMP6 and hepcidin mRNA, whereas the expression of NS3-NS5B proteins did not show such an increase (Figs 2F and S4). HCV Core-NS2- or GFP-expressing adenovirus (AdV) were injected into the tail veins of C57/BL6J (WT) and CREBH-KO (Creb3l3^−/−^) mice to analyze hepcidin and BMP6 expression in liver tissue. We found that HCV core-NS2 expression in WT mice led to increases in hepcidin and BMP6 mRNA expression, but no increase was observed in CREBH-KO mice, indicating that CREBH plays an important role in the induction of HCV-associated hepcidin expression, not only in cultured cells but also in liver tissues (Fig 2G). Since the co-existence of the N-terminal region of the NS3 protein is known to be required for the protease activity of HCV NS2, it is unlikely that CREBH is directly cleaved by NS2 protease activity in the case of Core-NS2 expression. Nevertheless, whether NS2 protease activity may be involved in the proteolytic cleavage of CREBH was tested. We co-expressed the full-length CREBH (CREBH-F) with Core-only, Core-NS2 or Core-NS2 C113S, which contains a mutation in the active center of the NS2 protease, and analyzed CREBH-F cleavage by western blotting (S5 Fig). The result showed that the cleaved fragment of CREBH (CREBH-N) was detected in the expression of Core-NS2 or Core-NS2 C113S more markedly compared to Core-only expression, suggesting that the activation of CREBH is not due to NS2 protease activity. Further analysis was performed to determine whether the induction of CREBH activation by Core-NS2 is due to the activation of general ER stress or unfolded protein response (UPR) which has been shown to activate CREBH [24]. Activation of the protein kinase R (PKR)-like endoplasmic reticulum kinase (PERK) pathway and inositol requiring enzyme-1 (IRE1) pathway was determined by detection of phosphorylation of eukaryotic translation initiation factor 2α (eIF2α) and splicing of X-box binding protein 1 (XBP1) mRNA, respectively (S6A and S6B Fig). Both phosphorylated eIF2α and XBP1 spliced RNA were induced by expression of HCV Core-NS2- or Core-NS2 C113S to the same level as in HCV infection. Their induced levels were clearly greater than that of Core expression alone. Activating transcription factor 6 (ATF6) is known to be a critical determinant of dynamics of transcriptional factor CCAAT-enhancer-binding protein homologous protein (CHOP) during UPR. The expression of Core-NS2- or Core-NS2 C113S resulted in a significant increase in CHOP mRNA expression, whereas the induction of CHOP expression was only limited in the case of Core expression alone (S6C Fig). These results suggest that HCV Core-NS2 is involved in the activation of general ER stress and its activation is independent of NS2 protease activity, and that Core-NS2 expression potentially induces the ER stress more efficiently compared to the case of Core-only expression.

### Decrease in serum levels of hepcidin-25 following viral clearance by antiviral therapy in hepatitis C patients

**Fig 3:**
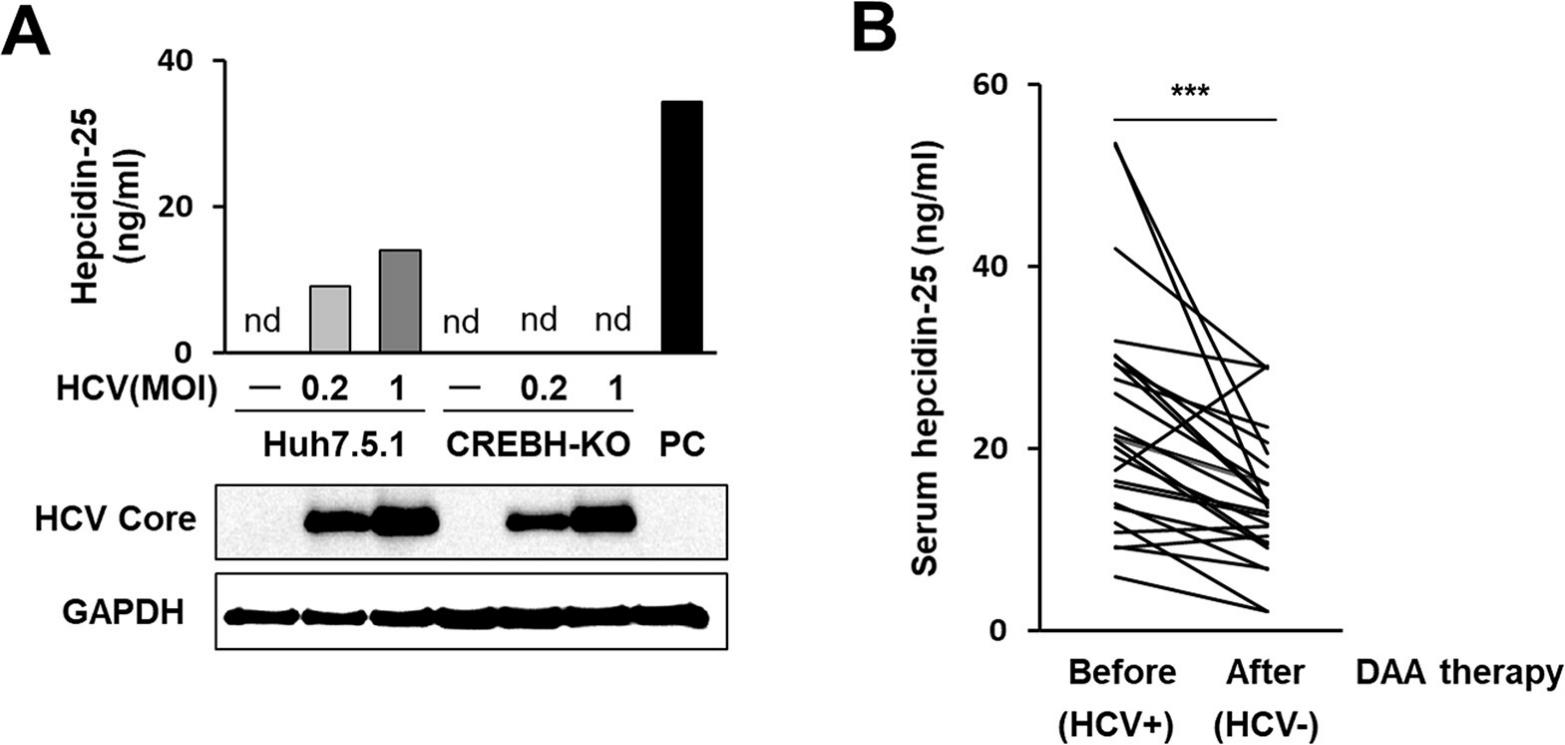
Hepcidin-25 measurement in culture supernatants with HCV infection and in sera from hepatitis C patients. (A) Hepcidin-25 concentrations in the supernatants of HCV-infected CREBH-KO cells and parental cells at 3 dpi at MOIs of 0, 0.2, and 1 were measured by the LC-MS/MS. As a positive control (PC), culture supernatants of cells transfected with the hepcidin-expressing plasmid were collected at 2 dpt and used. Expression of HCV Core and GAPDH in cells was assessed. (B) Changes in serum hepcidin-25 concentrations with decreasing viral loads in hepatitis C patients were analyzed. Paired serum samples from 26 hepatitis C patients were collected before (HCV positive) and after DAA therapy (HCV negative), and serum hepcidin-25 concentrations before vs. after viral elimination were compared. Significant differences between two groups before and after therapy were analyzed using Wilcoxon rank sum tests (***P<0.001).

To determine whether upregulation of hepcidin mRNA expression by HCV leads to changes in hepcidin-25 peptide levels, concentrations of hepcidin-25 in the culture supernatants of HCV-infected cells were measured by quantitative liquid chromatography tandem mass chromatography (LC-MS/MS). In Huh7.5.1 cell cultures, the hepcidin-25 level in culture supernatants increased in relation to the viral infection level. In contrast, in CREBH-KO Huh7.5.1 cell cultures in which the viral proteins were expressed as much as in the infected Huh7.5.1 cells, hepcidin-25 concentrations in the supernatants were below the detection limit even at a high infectious titer (MOI = 1) (Fig 3A).

Hepcidin has been identified as an acute phase protein induced by inflammatory factors, and it is not always clear how its expression changes in chronic inflammatory conditions. To address this issue and to investigate whether hepcidin-25 levels in human blood are associated with the presence or absence of HCV infection, we quantitated hepcidin-25 in pre-treatment (HCV positive) and post-treatment (HCV negative) serum of hepatitis C patients who had achieved sustained virologic response (SVR) by DAA therapy. Of the 26 paired patient sera measured, 23 patients showed a decrease in serum hepcidin-25 concentration due to SVR. A comparison of the pre-treatment and post-treatment groups for all 26 patients showed that serum hepcidin-25 levels were significantly lower in the post-treatment group (Fig 3B). To validate our methodology used, several patients’ sera that showed the hepcidin-25 concentration of approximately 5–50 ng/mL were analyzed by a company for clinical laboratory testing. The correlation analysis between the results of the analysis at the laboratory and the results of the measurement in our hand showed a high correlation (R^2^ = 0.9985), demonstrating that the hepcidin-25 measurement data performed in this study are reliable (S7 Fig). Demographic and clinical characteristics of hepatitis C patients with measured serum hepcidin-25 are shown in S1 Table. Our findings suggest that hepcidin expression levels can fluctuate with the loss of viral infection not only during acute inflammation but also during chronic inflammation such as hepatitis C.

### Proteolytic cleavage of FPN1 mediated by HCV NS3-4A protease

**Fig 4:**
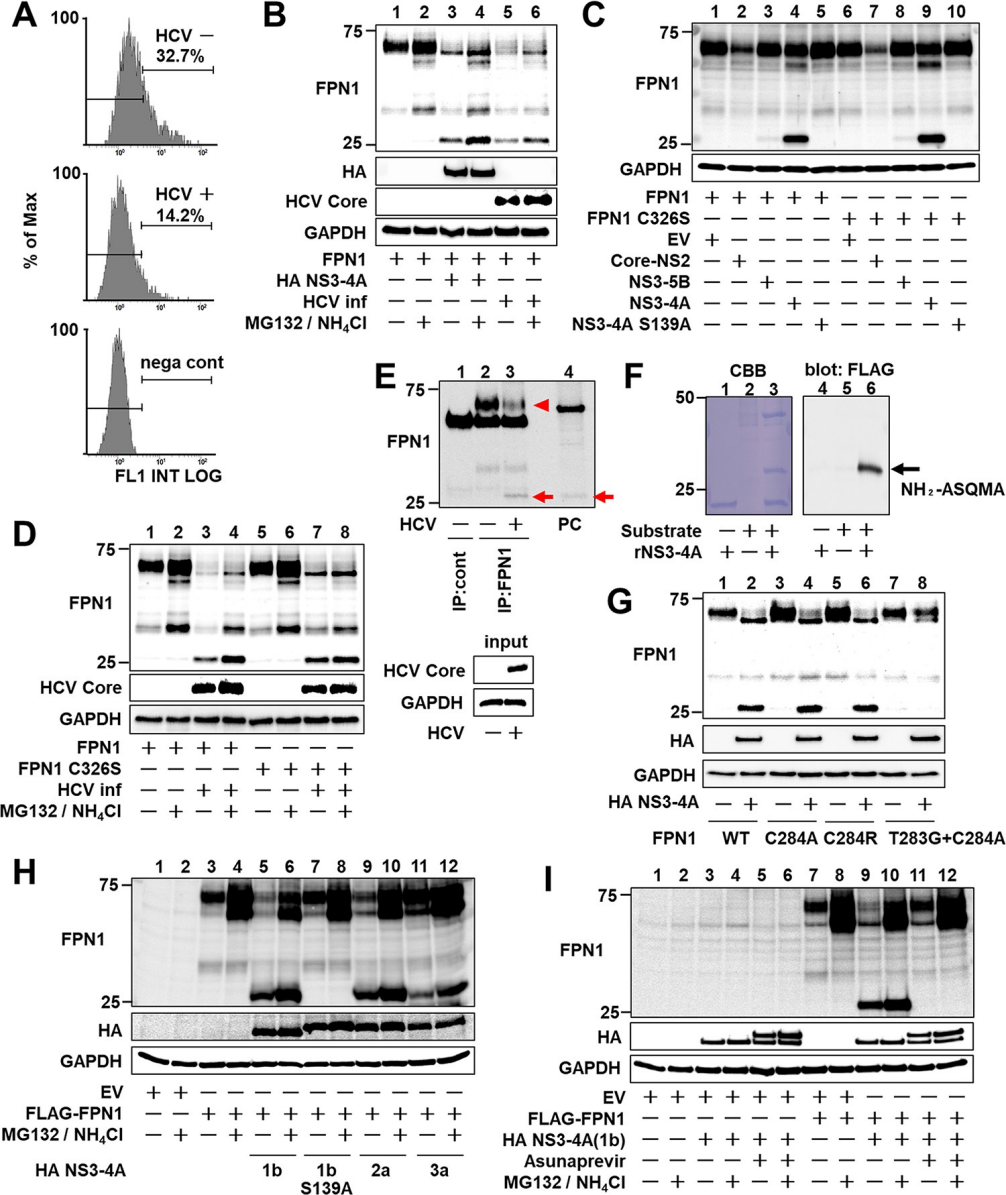
Proteolytic cleavage of FPN1 mediated by HCV NS3-4A protease. (A) Huh7.5.1 cells with or without HCV infection 1 day after transfection of the FPN1-expression plasmid were harvested at 1 dpi and reacted with anti-FPN1 antibody. The percentage of FPN1-positive cells was counted by flow cytometry. Cells transfected with an EV (nega cont) were used as a negative control. The figure is representative of three experiments with similar results. (B, D) At 1 dpi, cells with or without HCV infection were transfected (B) with the FPN1-expression plasmid together with the NS3-4A expression plasmid or the empty vector, (D) with plasmids expressing FPN1 or FPN1 C326S. At 2 dpt, FPN1, HCV Core, and GAPDH were analyzed by western blotting. (C) FPN1 and GAPDH were analyzed by western blotting with cell lysates transfected with both plasmids expressing FPN1 or FPN1 C326S and Core-NS2, NS3-5B, NS3-4A, or NS3-4A S139A at 2 dpt. (E) HCV-infected cells 3 dpi were immunoprecipitated with anti-FPN1 antibody (BMP033) or rabbit IgG, followed by western blotting using anti-FPN1 antibody (NBP1-21502). HCV Core and GAPDH in input samples were assessed by western blotting without immunoprecipitation. A cell lysate transfected with plasmids expressing FPN1 and NS3-4A was used as a positive control (PC). Endogenous full-length- and processed FPN1 are indicated as an arrowhead and arrow, respectively. (F) MBP-FPN1 (aa 278–292)-sfGFP-FLAG fusion protein as a substrate was reacted with recombinant NS3-4A. After SDS-PAGE and transfer to a PVDF membrane, the processed band stained with CBB (left) was excised, followed by N-terminal analysis. Processing of the substrate fusion protein was confirmed by western blotting using anti-FLAG antibody (right). (G-I) FPN1, HA-tagged NS3-4A, and GAPDH were analyzed by western blotting. Cells were transfected with (F) the plasmid expressing WT- or mutated FPN1 together with the expression plasmid for HA-tagged NS3-4A or EV, (H) the FLAG-FPN1-expressing plasmid together with the HA-NS3-4A expressing plasmid from genotypes 1b, 2a, or 3a, and (I) the plasmids expressing FLAG-FPN1 and HA-NS3-4A in the presence or absence of 1 μM asunaprevir. (B, D, H, I) MG132 and NH_4_Cl were added at final concentrations of 10 μM and 10 mM, respectively, 12 h before cell harvesting.

Hepcidin can bind to the iron transporter FPN1, and hepcidin-FPN1 binding triggers internalization, ubiquitylation, and degradation of FPN1. Therefore, high expression of the hepcidin gene and increased extracellular secretion of hepcidin peptide potentially lead to a reduced steady-state level of FPN1. Flow cytometry analysis of changes in cell surface FPN1 levels upon HCV infection showed that the intensity of FPN1 expression was decreased by HCV infection when FPN1-ectopically expressing cells that were infected with HCV were compared to those without infection (Fig 4A).

To further analyze the effects of HCV infection on the hepcidin-FPN1 system, we performed a sequence motif search that revealed the presence of a consensus cleavage site for HCV NS3-4A protease, D/E-X-X-X-X-C/T↓S/A (where X represents any residue), in the FPN1 protein. To determine whether FPN1 is actually cleaved by the expression of HCV NS3-4A, cells expressing FLAG-tagged FPN1 were generated, and the effect of HCV infection or viral protein expression was analyzed by western blotting. A cleaved form of FPN1 presumably corresponding to the N-terminal side of FPN1 was detected in HCV-infected cells (Fig 4B, lanes 5 and 6) and cells expressing NS3-S4A (Fig 4B, lanes 3 and 4; Fig 4C, lane 4) or NS3-S5B (Fig 4C, lane 3) derived from the viral Con-1 strain (genotype 1b). This band was not observed in cells expressing Core-NS2 or an active center mutant (S139A) of NS3-4A (Fig 4C, lanes 2 and 5). Such FPN1 cleavage by HCV infection was also observed in the case of FPN1 with a mutation in the hepcidin-binding site (C326S) (Fig 4D, lanes 7 and 8), suggesting that the proteolytic cleavage of FPN1 as mediated by HCV NS3-4A is hepcidin independent. The levels of full-length FPN1 and its cleaved form increased at least to some extent by the addition of lysosomal and proteasomal inhibitors (Fig 4B and 4D) and confirmed that the FPN1 cleaved protein produced by HCV NS3-4A expression and that caused by HCV infection are the same molecular size (Fig 4B). Since the anti-FPN1 antibody used in this study recognizes the N-terminal region of FPN1, the C-terminal FPN1 fragment is not detected by western blotting as shown in Fig 4. To detect both the N-terminal and C-terminal fragments of FPN1 cleaved by HCV NS3-4A, we created a new plasmid expressing FLAG-FPN1-myc protein with a FLAG tag at the N-terminus and a c-myc tag at the C-terminus of FPN1. When this FLAG-FPN1-myc was co-expressed with NS3-4A or expressed in HCV-infected cells, the N-terminal fragment of FPN1 was detected with anti-FLAG antibody as the primary antibody, just as it was detected with the anti-FPN1 antibody. In contrast, the C-terminal fragment of FPN1 was detectable with an anti-c-myc antibody (S8 Fig). We confirmed that the observed FPN1 cleavage by HCV NS3-4A was not an artifact of ectopic expression of FPN1 (Fig 4E), because cleaved FPN1 protein not seen in uninfected cells was endogenously detected in HCV-infected cells at 3 days post-infection, and the level of full-length FPN1 decreased accordingly (lane 2 vs. lane 3). The band size of the cleaved form of FPN1 was the same as that observed by co-expression of NS3-4A and FPN1 (lane 4). Due to the limited sensitivity of detection by anti-FPN1 antibodies, immunoprecipitation-WB was performed in this experiment using two different anti-FPN1 antibodies. To identify the cleavage site of FPN1 by HCV NS3-4A, maltose-binding protein (MBP)-FPN1 (aa278-292)-superfolder GFP (sfGFP)-FLAG fusion protein, in which the FPN1 region containing the putative cleavage site was fused with MBP at the N-terminus and sfGFP-FLAG at the C-terminus, was expressed in *E*. *coli* and affinity purified. The FPN1-fusion protein obtained was cleaved with a recombinant NS3-4A protease *in vitro* (Fig 4F). The N-terminal sequence analysis of the cleaved product revealed that the cleavage site by NS3-4A is between Cys (aa284) and Ala (aa285) matched consensus sequence D/E-X-X-X-X-C/T↓S/A in the intracytoplasmic region of the central portion of the FPN1 protein (S9 Fig).

Next, we examined how mutations at the FPN1 cleavage site affect cleavage by NS3-4A. When a mutation was introduced at the P1 site of the cleavage site, both the C284A and C284R mutations were found to maintain cleavage by NS3-4A (Fig 4G, lanes 4 and 6). However, for T283G/C284A double-mutant FPN1, in which both the P1 and P2 sites were replaced, cleavage by NS3-4A was no longer observed (Fig 4G, lane 8). Since the P2 position of the FPN1 cleavage site is Thr (aa283), it is likely that a single mutation to the P1 position at aa284 results in the residue at aa283 becoming P1 and maintaining cleavage by HCV NS3-4A.

In the NS3-4A expression experiment described above, HCV genotype 1b was used; to determine whether FPN1 cleavage activity is also observed in other genotypes of HCV, FPN1 cleavage analysis was performed for NS3-4A from genotypes 2a (JFH-1 strain) and 3a (S52 strain) (Fig 4H, lanes 9 and 11). The results showed that FPN1 is also susceptible to NS3-4A from HCV genotypes 2a and 3a. As expected, FPN1 cleavage by HCV NS3-4A was completely inhibited by the addition of 1 μM of the HCV protease inhibitor asunaprevir (Fig 4I, lanes 11 and 12).

### NS3-4A-dependent cleavage of FPN1 in mouse liver tissues

**Fig 5:**
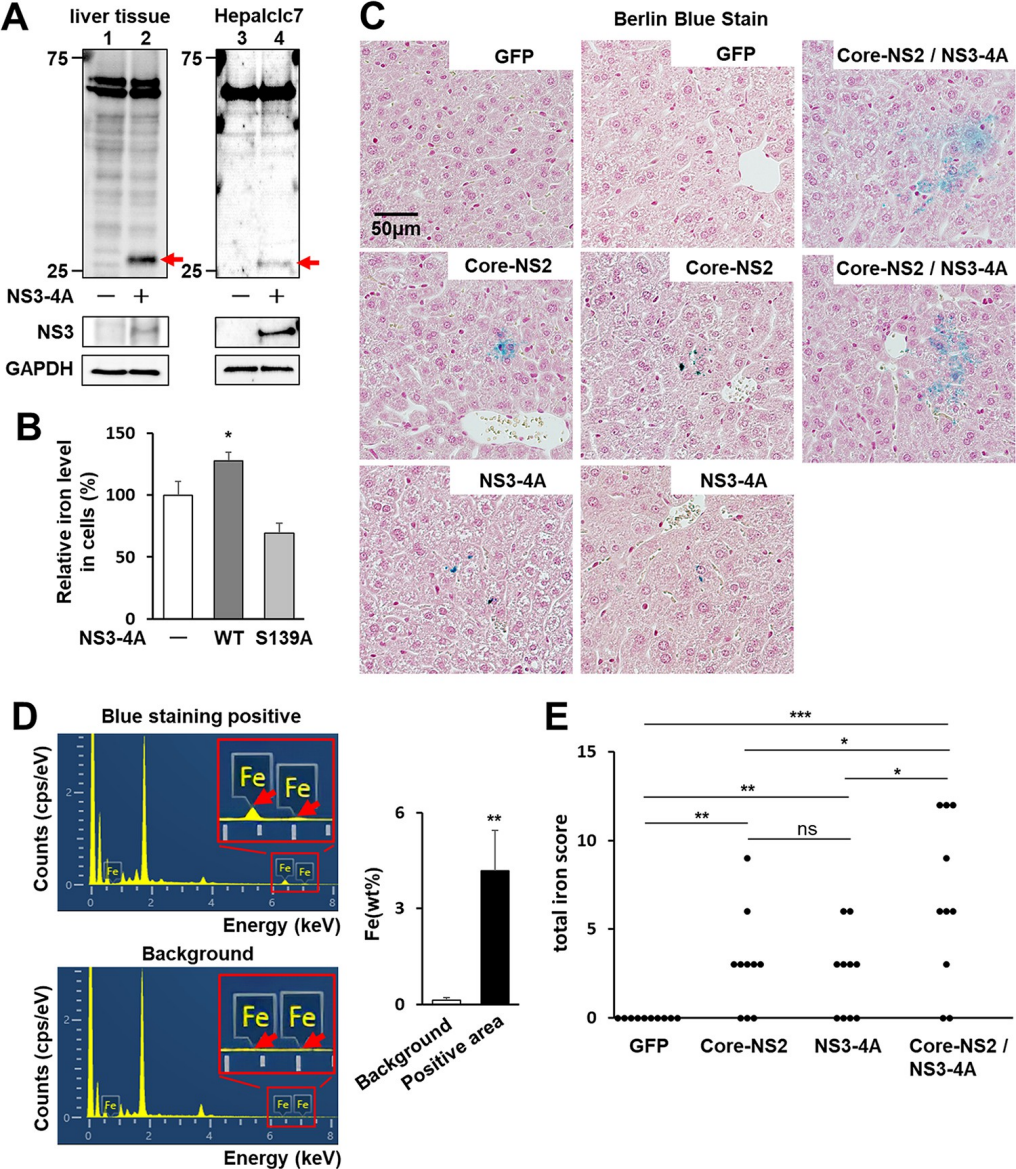
Iron store levels in mouse liver tissues and in cultured cells expressing HCV proteins. (A) Eight-week-old male WT mice were intravenously injected with AdV expressing either GFP or NS3-4A genotype 1b. At 5 dpi, liver tissues were subject to western blotting to detect FPN1 and its cleaved form (red arrow) and HCV NS3 (left). Hepalclc7 cells transfected with the NS3-4A-expressing plasmid or the EV (right). (B) Iron concentrations in cells transfected with the plasmid expressing FPN1 together with the expression plasmid for NS3-4A WT or S139A mutant or EV were measured using the PSAP-method. Mean values obtained from transfectants with the FPN1-plasmid and EV were set as 100%. Results represent the means with SD from three independent measurements. Student’s *t* test; *P<0.05, **P<0.01. (C) Four groups of mice (N = 4 each) were created in which recombinant adenoviruses were injected as follows: GFP group as a negative control; AdEFLNLGFP, AdEFNCre and AdEFGFP, Core-NS2 group; AdEFLNLHCVCore/NS2 and AdEFNCre, NS3-4A group; AdEFHCVNS3/4A, Core-NS2 and NS3-4A group; AdEFLNLHCVCore/NS2, AdEFNCre and AdEFHCVNS3/4A. Thin section slices were prepared from livers at 5 dpi and stained with Berlin blue. Representative images of two mouse livers from each group are shown. (D) Elemental analysis was performed with the NanoSuit method at the six spots positive for staining (Blue staining positive) and unstained parts (Background) in slices from the Core-NS2 group described in S13 Fig. (Left) Elemental composition graphs of energy (keV) vs. cps per eV are shown. Areas including the Fe peaks in the sample positive for blue staining and the corresponding area in the background sample are expanded. (Right) The weight concentrations of Fe measured were quantified and are expressed as weight percentages (wt%). Student’s *t* test; **P<0.01. (E) Total iron scores of 10 fields of view in the livers from the four groups described in (C) were individually evaluated by means of histological hepatic iron index. Results represent the plots separately for each group. Welch’s *t* test; *P<0.05, **P<0.01, ***P<0.001, ns P>0.05.

The hepcidin-FPN1 system is also conserved in mice. Human and mouse FPN1 show high amino acid sequence homology, and the sequence around the cleavage site by HCV NS3-4A is completely conserved (S3 Fig). In mouse hepatocellular carcinoma Heplclc7 cells, in which NS3-4A was ectopically expressed, a cleaved form of endogenous FPN1 was observed as expected (Fig 5A, lane 4). To demonstrate that FPN1 can be cleaved by NS3-4A not only in cultured cells but also in liver tissues, a recombinant adenovirus carrying the NS3-4A gene was intravenously injected into mice, and the liver tissues were subjected to western blot analysis. The result showed that the NS3-4A-expressing mice had a cleaved form of FPN1, in good agreement with the results obtained using the cultured cell system (Fig 5A, lane 2).

### Iron deposition in the liver tissues of mice expressing HCV proteins

Next, we used the Nitroso-PSAP method to analyze whether intracellular iron levels were altered by HCV NS3-4A and/or FPN1 expression: intracellular iron levels were decreased in cells with FPN1 overexpression compared to control cells transfected with an empty vector (S10 Fig, bars 1 and 3). In both cell types, HCV infection led to increases in cellular iron levels (S10 Fig, bars 2 and 4). Ectopic expression of HCV NS3-4A (wild type) increased iron levels in cells, whereas expression of the inactive NS3-4A mutant (S139A) did not; instead, NS3-4A S139A-expressing cells showed lower iron levels than in control cells (Fig 5B).

Finally, to analyze the iron deposition in liver tissues from HCV protein-expressing mice, we injected HCV Core-NS2-expressing AdV or NS3-4A-expressing AdV alone, or Core-NS2-expressing AdV and NS3-4A-expressing AdV together, and 5 days later analyzed liver tissues with Berlin blue staining. As expected, FPN1 cleavage was observed in the livers of NS3-4A-expressing mice (S11 Fig), and hepcidin and BMP6 mRNA expression levels were significantly higher in Core-NS2-expressing mice than in NS3-4A-expressing mice alone or negative control, GFP-expressing mice (S12 Fig). No positive staining with Berlin blue was observed in the negative control mice, but a few positive images were observed in mice expressing HCV Core-NS2 alone or NS3-4A alone. clustered Berlin blue-positive regions in the liver (Fig 5C and 5E). Independently of the experiment, we compared iron deposition in the liver of HCV Core-NS2-expressing mice versus core-NS2- and NS3-4A co-expressing mice and confirmed that Core-NS2 and NS3-4A co-expressing mice showed significantly higher iron deposition (S13 Fig). Since the HCV core-NS2-expressing liver tissues showed Berlin blue positivity but to a limited extent, trace elemental analysis was performed by Scanning Electron Microscopy/Energy Dispersive X-Ray Spectroscopy (SEM/EDS) combined with NanoSuit-Correlative Light and Electron Microscopy (CLEM) to verify whether iron was actually deposited in the stain-positive areas. The Berlin blue-positive areas clearly showed more iron-specific characteristic X-rays and higher mass percentages of iron compared with the background areas in liver tissues (Fig 5D). Based on the Berlin blue staining data, the iron deposition levels in the liver tissues of each mouse group were compared by total iron score (Fig 5E). Both HCV protein-expressing mouse groups showed significantly higher scores than the negative control group. HCV Core-NS2 and NS3-4A co-expressing mouse groups showed higher iron scores than the Core-NS2 expressing mouse group.

Together with other findings obtained in this study, we conclude that both transcriptional upregulation of hepcidin gene expression and proteolytic cleavage of FPN1 are involved in the HCV-induced increase in intracellular iron levels.

## Discussion

In this study, an increase in intracellular iron levels induced by HCV infection was demonstrated using two methods: a metallo-assay and luminescence measurement with a fluorescent probe (Fig 1B), suggesting that HCV infection contributes not only to increasing total iron stores, but also to enhancing hydroxyl radical production by the Fenton reaction. Reactive oxygen species catalyzed by divalent iron ions are known to be involved in the induction of inflammation and DNA damage, and have been suggested to be involved in the pathogenesis of chronic liver diseases including HCC.

We investigated whether HCV infection alters the gene expression of various factors that may be involved in the regulation of intracellular iron levels, and found that the upregulation of hepcidin expression upon infection was the most prominent among those tested (Fig 1C). Hepcidin is a peptide hormone produced in the liver that plays a central role in the regulation of iron metabolism. In the present study, not only were hepcidin mRNA levels induced in HCV-infected cells within a few days, dependent on titers and duration of infection, but also in a long-term infection culture system where cells can be cultured without passaging for up to 3 weeks after infection (Fig 1E) [19,20]. From hepcidin peptide analyses by quantitative LC-MS/MS, we found that hepcidin-25 levels in cell culture supernatants are increased by HCV infection (Fig 3A) and that hepcidin-25 levels in sera from hepatitis C patients are significantly decreased with HCV elimination by antiviral therapies (Fig 3B), reflecting the elevated expression of hepcidin mRNA due to HCV infection. It is noted that in this study, hepcidin levels after HCV elimination in most patients were at reported healthy blood levels (22.2 ± 12.3 ng/mL) [25].

Since hepcidin expression is known to be induced by intracellular inflammation, it makes sense that hepcidin expression is potentially increased by HCV infection. However, the molecular mechanism of induced expression by HCV infection is not fully understood. The hepcidin promoter region contains recognition sequences for several transcription factors such as SMAD, C/EBP (CCAAT enhancer binding protein), CREBH, and STAT [10–14]. In this study, ChIP analyses showed that binding of CREBH and SMAD to the hepcidin promoter is increased by HCV infection (Fig 2A). In addition, CREBH binding to the promoter region of BMP6, which upregulates the BMP/SMAD pathway, was also found to be enhanced by HCV infection (Fig 2D). We have previously reported that HCV infection triggers ER stress in infected cells, leading to activation of the ER-membrane anchored CREBH through intramembrane proteolysis, and that the activated form of CREBH transits to the nucleus, thereby recognizing the TGF-β2 promoter and acting to enhance TGF-β2 transcription [23]. These findings thus suggest that the activation of CREBH triggered by HCV infection not only directly contributes to the transcriptional activation of hepcidin but also acts through activation of the BMP/SMAD pathway induced by increased BMP6 expression to enhance hepcidin transcription. The pivotal role of CREBH in the elevated mRNA expression of BMP6 and hepcidin induced by HCV infection was clearly demonstrated in CREBH-KO experiments both in cell cultures and a mouse model (Fig 2E and 2G). Serum hepcidin levels were significantly higher in the HCV Core-NS2 expressing mice than in the control mice, which is consistent with that of hepcidin mRNA (S14A Fig). The serum iron levels of the Core-NS2 expressing mice were significantly lower than those of the control mice (S14B Fig). The results are consistent with the fact that hepcidin induction by HCV Core-NS2 resulted in decreased iron uptake from the intestinal tract and decreased iron release from the cells into the blood due to increased iron accumulation in hepatocytes.

Based on the protein analysis of FPN1, we found that FPN1 is cleaved by HCV NS3-4A serine protease activity in cultured human HCC cells. Amino acid sequencing identified the region between C283 and A284, which is located in the large intracytoplasmic loop between the sixth and seventh transmembrane sites from the N-terminal side in FPN1, as a cleavage site (Fig 4F). We demonstrated that the same site is actually cleaved by the protease activity of NS3-4A in HCV-infected cells (Fig 4B, 4D and 4E) and the mouse model (Fig 5A). The amino acid sequences of mouse FPN1 and human FPN1 are very similar, with 90% or higher amino acid sequence homology [26], and the amino acid residues around the cleavage site by HCV NS3-4A are completely identical in both FPN1 proteins. The decrease in FPN1 level on the surface of HCV-infected cells observed in the flow cytometry analysis (Fig 4A) likely involves both the molecular mechanisms of HCV infection-induced enhancement of hepcidin expression, which promotes FPN1 degradation, and NS3-4A-mediated cleavage of FPN1.

It is noted that NS3-4A expression has no effect on mRNA expression of FPN1 and hepcidin (S15 Fig). To further clarify whether hepcidin and FPN1 affect HCV replication, cells with knockdown of hepcidin or FPN1 gene were used for HCV infection. No apparent influence on HCV propagation was observed in the case of either knockdown (S16 Fig), suggesting that the expression of hepcidin and FPN1 does not have at least a significant effect on HCV replication.

It has been reported that the HCV NS3-4A protease can proteolytically cleave several human proteins such as IPS-1/MAVS/VISA/Cardif [27,28], TRIF [29,30], OCIAD1 [31], DHCR24 [32], C4 [33], TC-PTP [34], DDB1 [35], and GPx8 [36] in addition to processing the viral precursor polyprotein. However, this is the first example of a host cellular substrate involved in the regulation of metal metabolism. In FPN1 with 12 transmembrane domains spanning the membrane and the N- and C-termini located on the intracellular side, 6 helices at the N-terminal side and 6 helices at the C-terminal side form lobe-like structures, respectively, and both lobes flexibly interact with each other. Structural modeling indicates that the conformational change of FPN1 from an inward to outward state upon Fe^2+^ binding is responsible for iron efflux from the cell [37,38]. It is likely that proteolytic cleavage at the intracellular loop between the 6th and 7th transmembrane helices mediated by HCV NS3-4A alters the mode of interaction of the two lobes, thereby interfering with the mobility of the intracellular gate and reducing the efficiency of iron transport. Next, we investigated whether FPN1 cleavage by HCV NS3-4A affects intracellular ion levels in the cultured cell line and mouse model and obtained the following findings. 1) The expression of wild-type NS3-4A in Huh7.5.1 cells increased intracellular iron levels, whereas no such increase was observed in the inactive NS3-4A mutant (Fig 5B), and 2) increased iron deposition was observed in the liver tissue of mice inoculated with recombinant adenoviruses carrying the NS3-4A gene (Fig 5C). These results support the working hypothesis that cleavage of FPN1 mediated by HCV protease impairs the iron efflux transporter function of FPN1, leading to an increase in intracellular iron levels. Although ubiquitin-dependent degradation of FPN1 induced by hepcidin binding is a well-known mechanism to regulate FPN1 function, this study shows for the first time that FPN1 cleavage by serine protease activity can also serve as a post-translational mechanism to negatively regulate iron efflux.

**Fig 6:**
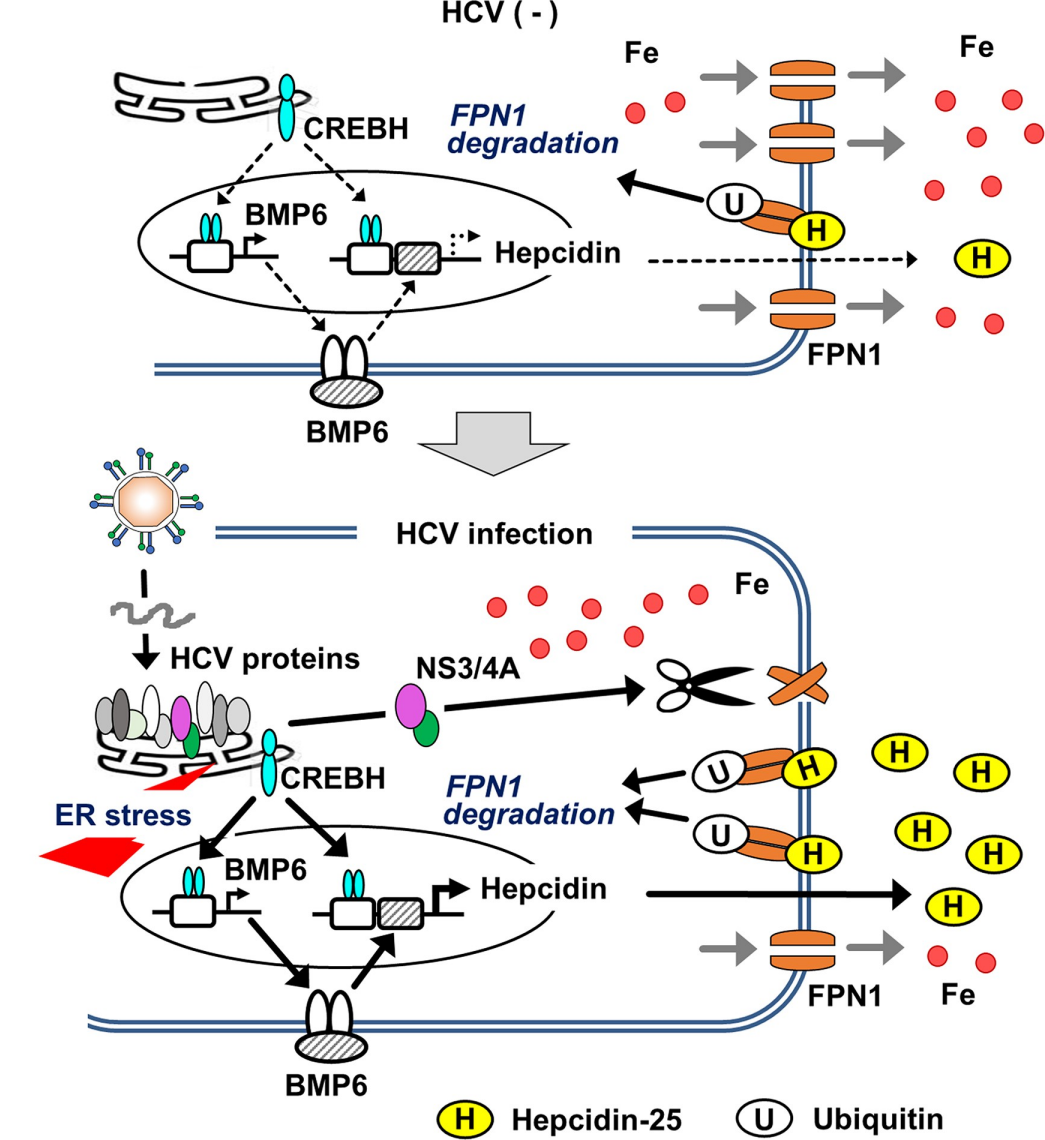
Working model of iron accumulation induced in HCV-infected hepatocytes. Much of the replication process of HCV is dependent on the ER of the host cell; in HCV-infected cells, a viral precursor polyprotein is cleaved into 10 proteins by ER-resident peptidases and viral proteases associated with the ER membrane. ER-derived membranes also play an important role in viral replication complex formation. These characteristics of the HCV lifecycle induce ER stress in infected cells. This activates the ER resident transcription factor CREBH, which in turn upregulates the transcription both of BMP6 and hepcidin through its recognition of the promoters. The induction of BMP6 expression enhances BMP signaling, and the Smad complex, which is activated as a result, also plays a role in activation of the hepcidin promoter. The induction of hepcidin gene expression leads to an increase in extracellular hepcidin peptide levels, which in turn increases hepcidin-FPN1 association, thus promoting retrograde transport of FPN1 into the cell and ubiquitin-dependent degradation. The NS3-NS4A complex, whose serine protease activity is essential for processing the viral precursor, can cleave the intracytoplasmic portion of the central region of FPN1, which is presumably suppressed in its iron efflux function upon cleavage.

In mouse experiments, iron deposition in liver tissue was more pronounced when co-infected with recombinant adenoviruses expressing Core-NS2 and NS3-4A, respectively, than when inoculated with Core-NS2 or NS3-4A expressing adenovirus alone (Fig 5C), which was expected to induce hepcidin expression from ER stress induction. Thus, these results suggest that both molecular mechanisms of transcriptional upregulation of the hepcidin gene upon infection and FPN1 cleavage by NS3-4A protease are potentially involved in iron accumulation in HCV-infected cells. Based on these findings, we constructed a model diagram depicting the mechanism by which iron levels in hepatocytes increase from HCV infection (Fig 6). ER stress generated by HCV replication in the infected cells activates the BMP-SMAD pathway through activation of the transcription factor CREBH, leading to upregulation of BMP6 gene expression. Increased binding of both CREBH and SMAD to the hepcidin promoter region markedly induces hepcidin transcription. The elevation of hepcidin peptide levels in response to the increased mRNA expression enhances hepcidin binding to FPN1, which in turn leads to FPN1 degradation. In addition, the serine protease activity of the NS3-4A complex produced through HCV replication cleaves the intracytoplasmic loop region of FPN1, resulting in a reduction of iron export by FPN1.

Although Flisiak et al. have shown that serum hepcidin levels tend to decrease in hepatitis C patients after DAA treatment [39], most studies reported to date have shown that HCV infection acts in a suppressive manner on hepcidin expression [15–18] and that chronic HCV infection leads to reduced hepcidin level in patients’ sera [40–46]. This mainstream finding of suppression of hepcidin production induced by HCV can nicely explain why chronic hepatitis C patients eventually develop iron overload, which is reflected in high serum iron and ferritin values, high transferrin saturation, and presence of histologically detectable iron deposits in the liver tissues. In contrast, the increased hepcidin expression due to HCV demonstrated in this study raises questions of physiological significance because it does not appear to reflect the findings seen in hepatitis C patients, as it may lead to hypoferremia. Hepcidin is inherently involved in the homeostasis of blood iron levels in the organism. Decreased serum iron causes hepcidin levels to drop, and iron flows into the blood from both hepatocytes and the intestinal tract. Within HCV-infected individuals, both the effects of HCV on the hepcidin-FPN1 system and the regulation of physiological iron metabolism are assumed to be at work. It might be hypothesized that the initial event after HCV infection is an increase in hepcidin expression and iron sequestration in infected cells, followed by a reduction in the induction of hepcidin expression by HCV and a tendency for hepcidin expression to be suppressed to compensate for intracellular iron excess. To determine whether the iron overload and hepcidin deficiency state in the body ultimately seen in persistent HCV infection can be triggered by iron overload in infected hepatocytes, a transgenic mouse model is being constructed in which HCV Core-NS2- and NS3-4A expression is induced by the Cre/lox system. Using this animal model, we plan to analyze changes in hepcidin expression and systemic iron levels over time from immediately after induction of the viral protein expression to several months.

In the era of DAA therapy, HCV elimination can now be achieved in patients with advanced chronic hepatitis C and in elderly patients who were previously ineligible for treatment. However, a significant number of patients with undetectable levels of HCV infection still remain at risk of hepatic carcinogenesis because of their long-term liver damage. Thus, although HCV-associated liver carcinogenesis is still an important public health issue, the molecular mechanisms underlying the development of HCC in hepatitis C patients remain poorly understood.

In this study, we found a critical role for ER stress induction and viral protease activity in iron accumulation in HCV-infected cells. Future studies will need to elucidate the contribution of hepatic iron accumulation to the development of liver failure and HCC in chronic hepatitis C pathology.

## Materials and methods

### Ethics statement

The experiments using clinical samples were approved by the Ethics Committee of Hamamatsu University School of Medicine. Informed consent in writing was obtained from each patient. Animal husbandry procedures and experiments using WT and Creb3l3^−/−^ mice were performed in accordance with the Regulations for Animal Experiments of Hamamatsu University School of Medicine and were approved by the Animal Experiment Committee of the university.

### Reagents

Human hepcidin-25 peptide and MG-132 (Peptide Institute, Osaka, Japan) and HCV NS3 protease inhibitor, asunaprevir (BMS-650032) were used.

### Cell culture

The mouse hepatoma line Hepa1c1c7, human hepatoma HepG2 cells, and HuH-7 and its derivative cell line Huh7.5.1, which is HCV negative and highly permissive for HCV infection [47] (a gift from Francis V. Chisari, The Scripps Research Institute, La Jolla, CA, USA) were grown essentially as described [48]. To arrest proliferation of hepatoma cells and allow the culture to continue for several weeks without passaging, Huh7.5.1 cells were cultured in Dulbecco’s modified Eagle medium containing 1% dimethyl sulfoxide (DMSO) [19] for 14 days before being used for HCV infection [20]. CREBH-KO cells derived from Huh7.5.1 cells were produced using the CRISPR-Cas9 system as described [22].

### Plasmids

pcDNA-FPN1, which expresses full-length FPN1 (1,716 amino acids), was constructed by inserting PCR products of corresponding FPN1 regions, which were amplified with Huh7.5.1-derived cDNA as a template, into pcDNA3.1 (Thermo Fisher Scientific, Waltham, MA, USA). To make expression plasmids for mutated FPN1 with substitutions and partial deletions, the GENEART Site-Directed Mutagenesis System (Thermo Fisher Scientific) and the Dpn I method (New England Biolabs, Ipswich, MA, USA), respectively, were used. To construct pcDNA-FLAG-FPN1, a PCR product obtained with a sense primer designed to contain the FLAG tag at the N-terminus of FPN1 and pcDNA-FPN1 as a template was inserted into pcDNA3.1. To create pcDNA-hepcidin, which is a hepcidin-expressing plasmid, a PCR product corresponding to the cDNA of the entire preprohepcidin region, which was amplified as per above, was inserted into pcDNA3.1. To construct promoter reporters for hepcidin, 2.7 kb region upstream of the translation initiation site of the hepcidin gene was obtained by PCR and inserted into pGL4.10 (Promega, Madison, WI, USA), yielding pGL-hepcidin pr. The BMP6 promoter reporter pGL-BMP6pr was constructed by inserting the BMP6 promoter sequence derived from pKM2L-phBMP6 (RDB05474), which was provided by RIKEN BRC through the National Bio Resource Project of MEXT, Japan, into pGL4.10. Expression plasmids for the HCV proteins pCAG-Core-NS2 were previously described [49].

pCAG-HACon1NS3NS4A, which expresses full-length NS3 and NS4A (derived from HCV genotype 1b, Con-1 strain) with an HA tag at the N-terminus of NS3, and its mutated construct pCAG-HACon1NS3NS4A S139A that has a point mutation of serine to alanine at position 139, were previously described [33]. To construct pCAG-HAJFH1NS3NS4A and pCAG-HAS52NS3NS4A, PCR fragments encoding the NS3-NS4A region derived from the JFH-1 strain (genotype 2a) and S52 strain (genotype 3a) [50], respectively, were digested with XhoI-NotI, followed by replacement with the corresponding part of pCAG-HACon1NS3NS4A. To construct pCAG-HACon1NS3NS5B, PCR fragments encoding the NS3-NS5B region derived from the Con-1 strain were replaced with the corresponding part of pCAG-HACon1NS3NS4A using In-Fusion HD Cloning Kit (Takara Bio Inc., Shiga, Japan).

### Virus infection and DNA transfection

HCV stock derived from J6/JFH-1 was prepared as previously described [51]. Naive Huh7.5.1 cells were infected with HCV J6/JFH-1 at various MOI for 4 h. Lipofectamine LTX (Thermo Fisher Scientific) was used for DNA transfection.

### RNA extraction and RT-PCR

Total cellular RNAs isolated by the TRI reagent (Molecular Research Center, Cincinnati, OH, USA) were transcribed using the SuperScript VILO cDNA Synthesis Kit (Thermo Fisher Scientific). Aliquots of cDNAs were subjected to 40 cycles of PCR amplification. Primer sequences used are shown in S2 Table. Quantitative (q)PCR was performed with the CFX Connect Real-Time System (Bio-Rad, Hercules, CA, USA) using THUNDERBIRD SYBR qPCR mix (Toyobo, Osaka, Japan). Briefly, reverse-transcribed cDNAs together with 6 pmol of forward and reverse primers were used for PCR. The thermal cycling conditions were: 1 min at 95°C, followed by 40 cycles at 95°C for 15 s and 60°C for 30 s. The RNA expression data were normalized to mRNA levels of β-actin or GAPDH using the comparative threshold cycle method.

### Luciferase reporter assay

Cells seeded in a 24-well plate at a density of 5 × 10^4^cells/well were transiently co-transfected with *Renilla* luciferase and firefly luciferase vectors with or without HCV infection. For conditions with HCV infection, the hepcidin- or BMP6-promoter reporter vectors were introduced into the cells at 1 dpi of Huh7.5.1 cells (MOI = 0.5). After 2 days, luciferase activities were measured using the Dual-Luciferase reporter assay system (Promega) and a Synergy H1 Hybrid Multi-Mode Microplate Reader (Biotek, Winooski, VT, USA). Each firefly luciferase activity was normalized to the *Renilla* luciferase activity expressed from pGL4.73 or pGL4.74 (Promega).

### Flow cytometry analysis

Huh7.5.1 cells transfected with pcDNA-FPN1 with or without HCV infection were collected with a scraper, followed by centrifuging at 500 × *g* for 3 min. The cells were incubated with the primary antibody (anti-SLC40A1 (ab85370)) for 1 h. After washing three times with PBS, the cells were incubated with a secondary antibody (anti-rabbit Alexa 488) for 1 h. Samples were measured with a Gallios Flow Cytometer (Beckman Coulter, Inc., Brea, CA, USA) and analyzed with FlowJo FCM analysis software.

### Immunoprecipitation and western blotting

Cells were washed with PBS and lysed for 5 min on ice in RIPA buffer (25 mM Tris-HCl pH 7.6, 150 mM NaCl, 1 mM EDTA, 1% Nonidet P-40, 1% sodium deoxycholate, 0.1% SDS) with a protease inhibitor cocktail (Roche Diagnostics, Rotkreuz, ZG, Switzerland). Lysates were cleared by centrifugation, and protein concentrations were determined using the BCA Protein Assay Reagent (Thermo Fisher Scientific). For tissue samples, liver tissues were homogenized with RIPA buffer with a protease inhibitor cocktail. Immunoprecipitation was performed by incubating cell lysates for 2 h at 4°C with the appropriate antibody and then for 2 h at 4°C with Protein G Mag Sepharose (Cytiva, Tokyo, Japan). Immunoprecipitates were washed three times in RIPA buffer and subjected to SDS-PAGE. Samples were treated with a 3-fold volume of 10 M urea at 37°C for 15 min prior to SDS-PAGE. Following SDS-PAGE of samples with equal protein contents, the proteins on the gels were transferred to a polyvinylidene difluoride membrane. After blocking for 1 h, the membranes were incubated with first antibodies for 1 h at room temperature. After washing, signals were detected using horseradish peroxidase-conjugated antibody (Cell Signaling Technology, MA) and chemiluminescence (GE Healthcare Japan, Tokyo, Japan). The antibodies used in this study are listed in S3 Table.

### Chromatin immunoprecipitation

The ChIP assay was performed with the SimpleChIP Enzymatic Chromatin IP Kit (Cell Signaling Technology, Danvers, MA, USA) according to the manufacturer’s protocol. Nuclear extracts were immunoprecipitated with a polyclonal anti-CREBH antibody (Affinity Biosciences, Cincinnati, OH, USA), monoclonal anit-Smad1 antibody (Cell Signaling Technology), or normal rabbit IgG (Cell Signaling Technology) as a negative control. DNA samples prepared from the precipitates were subjected to qPCR using primers encompassing the human hepcidin and BMP6 promoter as shown in S2 Table.

### Metallo assay

Cells seeded in a 12-well plate at a density of 1 × 10^5^ cells/well were transiently co-transfected with pcDNA-Fpn1 and pCAG-HANS3NS4A or -NS3NS4A S139A. After 2 days, intracellular iron ions (Fe^2+^ and Fe^3+^) in cell lysates were measured with the Metallo Assay Iron Assay Kit LS Nitroso-PSAP method (Metallogenics, Chiba, Japan). For HCV infection experiments, cells were infected with HCV J6/JFH-1 at various MOI for 4 h, and intracellular iron ions were measured at 3 dpi.

### Detection of Fe^2+^ using the Labile Ferrous Ion Detecting Probe FeRhoNox-1

Intracellular iron Fe^2+^ ions via orange (red) fluorescence after treatment with the activatable fluorescent probe FeRhoNox-1 (GORYO Chemical, Hokkaido, Japan) were specifically observed with a laser scanning microscope, FV-1000 (OLYMPUS, Tokyo, Japan), for confocal microscopic analysis. Cytoplasmic luminescence intensity was measured using Image J software 1.47v (National Institutes of Health, Bethesda, MD, USA).

### Quantification of Hepcidin-25 by LC-MS/MS analysis

Synthetic human hepcidin (DTHFPICIFCCGCCHRSKCGMCCKT) and stable isotope-labeled (SI) human hepcidin (DTH[^13^C_9_, ^15^N]FPICI[^13^C _9_, ^15^N]FCC[^15^N]GCCHRSKCGMCCKT) as an internal standard (IS) were purchased from the Peptide Institute. The hepcidin and IS stock solutions (100 μg/mL) were generated by diluting 50/50/0.1 (v/v/v) with water/ethanol/formic acid and were stored at −20°C in Protein LoBind Tubes (Eppendorf, Hamburg, Germany). Working solutions of hepcidin and IS were prepared by diluting stock solutions at 2 μg/mL and 1 μg/mL in 50/50/0.1 (v/v/v) acetonitrile (ACN)/water/formic acid, respectively. Standard samples were obtained by serial dilutions of the working solution.

For measuring hepcidin concentrations in culture supernatants of HCV-infected cells, equal amounts of 4% TCA and internal standard working solution were added to 10 mL of HCV-infected culture supernatants and centrifuged at 9,500 x g for 25 min. After removing the supernatants, the precipitates were dissolved in 200 μL of 40/60/1 (v/v/v) ACN/water/formic acid and filtered through a 0.20-μm polytetrafluoroethylene (PTFE) membrane.

To determine the hepcidin level in hepatitis C patient sera, sample preparations from sera were performed using Oasis HLB cartridges (Waters, Milford, MA, USA). The manufacturer’s protocol was optimized as follows. Briefly, 200 μL human serum was diluted v/v with 0.1% formic acid solution after adding the internal standard working solution. After a vortex step for 10 s and centrifugation, supernatants were entirely transferred into the Oasis HLB, which was pretreated with 1 mL of methanol and 1 mL of water under vacuum. The cartridges were washed sequentially with 1 mL of 75/30/5 (v/v/v) water/methanol/5% ammonium hydroxide. The extracts were eluted by 1 mL of 10/90/0.1 (v/v/v) water/methanol/formic acid and evaporated at 43°C for 3 h with a Savant SpeedVac Plus SC110A concentrator (SAVANT Instruments, Holbrook, NY, USA). After evaporation, samples were dissolved in 200 μL of 40/59/1 (v/v/v) ACN/water/formic acid and filtered through a 0.20-μm PTFE.

The LC system consisted of an Acquity ultra performance liquid chromatography (UPLC) unit (Waters). The mobile phases were 0.1% formic acid in water (A) and 0.1% formic acid in ACN (B), and the flow rate was 0.3 mL/min. After injection, mobile phase B was linearly ramped from 15% to 35% in 5 min, then reached 100% in 2 min. The proportion of B was maintained at 100% for 3 min and was then reduced rapidly to 15% for 0.1 min for re-equilibrating. The run duration of each sample lasted 18 min. The MS/MS analysis was performed in positive ESI mode using a 4000Q-TRAP quadropole linear ion-trap hybrid mass spectrometer (AB SCIEX, Tokyo, Japan) with the following settings: ion spray voltage, 4500 V and temperature, 600°C. Hepcidin was detected by multiple reaction monitoring. The ions were selected in the first quadrupole (Q1) and collided with nitrogen gas in the second quadrupole (Q2), and the product ions were detected in the third quadrupole (Q3). Human hepcidin was quantified by using the ion transition 698.3 (Q1) → 109.9 (Q3) for the non-labeled hepcidin and 563.0 (Q1) → 69.9 (Q3) for IS.

### Recombinant adenoviruses

The cDNA encoding HCV Core-NS2 derived from the JFH-1 strain was cloned into the cosmid cassette of pxAdEFLNLw-FVF containing the full-length AdV genome. The Cre recombinase inducible AdV (AdEFLNLHCVCore/NS2) and Cre-expressing AdV (AdEFNCre) were prepared as previously described [52]. The AdV vector AxEFLNLG as a control of Cre efficiency and the purification method were as previously described [53]. To produce the AdV vector expressing HCV NS3-4A (AdFEHCVNS3/4A), the cDNA encoding the HCV NS3-NS4A region of JFH-1 was cloned into the cosmid cassette of pAxEFwit2 [54], which contains the EF1alpha promoter and the full-length AdV genome excluding E1 and E3 coding regions. AdFEHCVNS3/4A and AdV expressing GFP (AdEFGFP) as a control were prepared [54].

### Mouse experiments

Male C57/BL6J (WT) mice were obtained from SLC Japan (Hamamatsu, Japan), and the CREBH null-mutant, Creb3l3tm1.1Sad/J (Creb3l3^−/−^) mice were a kind gift from Prof. Hitoshi Shimano, Faculty of Medicine, University of Tsukuba, Japan [55]. For AdV infection, eight-week-old male mice were infected with AdEFNCre [53] together either with AdEFGFP or AdEFLNLHCVCore/NS2 at 1.0 × 10^9^ pfu per mouse, followed by collecting liver samples 5 dpi. WT mice were infected either with AdEFGFP or AdFEhHCVNS3/4A at 2.0 × 10^9^ pfu per mouse, followed by collecting liver samples at 5 dpi. For co-expression of HCV C-NS2 and NS3-4A, eight-week-old male mice were infected with AdEFNCre and AdEFLNLHCVCore/NS2 at 1.0 × 10^9^ pfu per mouse, together with AdFEHCVNS3/4A at 2.0 × 10^9^ pfu per mouse, followed by collecting liver samples at 5 dpi. Iron deposits were assessed by scoring according to their size and their cellular and lobular locations in hepatic lobules. Total iron scores were calculated from the sum of hepatocytic, sinusoidal, and portal iron scores as previously described [56].

### NanoSuit-CLEM method combined with SEM-EDS

The preparation of specimens for observation with the NanoSuit method was previously described [57]. Spots stained with Berlin blue and unstained parts on the slides of mouse liver tissues were investigated by SEM observation using CLEM-EDS and the NanoSuit method as previously described [58,59]. Elemental analysis of iron was performed with Low-vacuum SEM (Lv-SEM) (TM4000Plus; HITACHI, Tokyo, Japan; accelerating voltage: 15 kV) equipped with an EDS (X-stream-2; Oxford instruments, Oxford, UK). AZtecOne software (Oxford Instruments) was used for the EDS analysis. The weight concentrations of detected elements were quantified and expressed as weight percentages (wt %), which shows the relative concentration of an element in the analyzed area.

### Statistical analyses

All statistical analyses were conducted using IBM SPSS Statistics version 25 (IBM, Armonk, NY, USA). Values are expressed as means with standard deviation (SD). Statistical significance was calculated using an unpaired Student’s *t* test, Welch’s *t* test or Mann-Whitney rank sum test. Statistical significance was set at P<0.05.

## Supporting information

S1 Text Supporting Materials and Methods. (DOCX)

S1 Fig Ferritin protein levels in HCV-infected cells. The protein levels of ferritin in cells with or without HCV infection as described Fig 1C were detected by western blotting. Intensities of the protein bands were quantified by ImageJ.
(TIF)

S2 Fig Induction of hepcidin mRNA expression by addition of concentrated culture media of cells with or without HCV infection. Concentrates of culture supernatants of Huh7.5.1 cells with or without HCV infection were prepared by the same ultrafiltration method used for the preparation of HCV stock. After 3 days of inoculation with the concentrate from HCV-infected cells (+) or uninfected cells (-) to naïve Huh7.5.1 cells, intracellular hepcidin mRNA levels were analyzed using qRT-PCR. Results represent the means with SD from three independent measurements. Student’s t test; ***P <0.001.
(TIF)

S3 Fig Involvement of BMP6 in hepcidin mRNA induction. (A) Recombinant BMP6 at a final concentration of 100 ng/mL (+) or vehicle (-) was added to Huh7.5.1 cells. After 48 h, total RNAs were prepared, and hepcidin mRNA was analyzed by qRT-PCR. (B) Cells were transfected with a plasmid expressing BMP6 or an empty vector (EV). At 2 dpt, total RNAs were extracted from cells, and mRNAs of hepcidin and GAPDH were quantified by qRT-PCR. (C) siRNA targeted to BMP6 (Silencer Select siRNA, s2032) or a universal negative control siRNA (sicont) at 10 nM was introduced into Huh7.5.1 cells. At 2 dpt, total RNAs were extracted from cells, and mRNAs of hepcidin and BMP6 were quantified by qRT-PCR. Results represent the means with SD from three independent cultured cell samples. Student’s t test; **P<0.01, ***P<0.001.
(TIF)

S4 Fig Induction of hepcidin and BMP6 mRNA expression in HuH-7 and HepG2 cells upon overexpression of HCV Core-NS2 protein. HCV Core-NS2 or GFP-expressing plasmid was transfected into HuH-7 cells or HepG2 cells. At 2 dpt, total RNAs were extracted from cells, and mRNAs of hepcidin and BMP6 were quantified by qRT-PCR. Student’s *t* test; *P<0.05, **P<0.01, ***P<0.001.
(TIF)

S5 Fig CREBH activation by HCV protein expression. (A) Cells were transfected with a plasmid expressing the full-length FLAG-tagged CREBH (CREBH-F) together with FLAG-tagged HCV Core, HCV Core-NS2 and HCV Core-NS2 C113S expressing vector or the empty vector (EV). At 2 dpt, proteolytically-processed activated CREBH (CREBH-N) was analyzed by western blotting. Cells transfected with the plasmid expressing the FLAG-tagged CREBH-F (red arrowhead) together with FLAG-tagged CREBH-N (red arrow) were used as a control. Open triangles, nonspecific proteins detected. (B) indicates the original data used for (A).
(TIF)

S6 Fig HCV Core-NS2 protein expression or HCV infection activates signaling pathways of the unfolded protein response (UPR). (A) Huh7.5.1 cells were transfected with the FLAG HCV Core, HCV Core-NS2, HCV Core-NS2 C113S expression plasmid or the empty vector (EV). The cells with HCV infection were also prepared. After 3 days of transfection or infection, eukaryotic initiation factor 2α (eIF2α), p-eIF2α, HCV Core and GAPDH were analyzed by western blotting. (B-C) Total RNAs from cells as described in (A) were extracted by the TRI reagent (Molecular Research Center, Cincinnati, OH, USA) and were transcribed using the SuperScript VILO cDNA Synthesis Kit (Thermo Fisher Scientific). (B) The splicing of x-box binding protein 1 (XBP1) mRNA was detected by RT-PCR using specific primers. PCR products were separated by electrophoresis on 2% agarose gels and visualized by ethidium bromide staining. (C) mRNA expression of CCAAT enhancer-binding protein (C/EBP)-homologous Protein (CHOP) was analyzed using qRT-PCR. Results represent the means with SD from three independent measurements. Student’s *t* test; **P<0.01, ***P <0.001.
(TIF)

S7 Fig Comparison of serum hepcidin concentration in hepatitis C patients measured by our LC-MS/MS protocol and measured by a company for clinical laboratory testing. Sera from five hepatitis C patients before DAA therapies were used. Serum hepcidin concentrations were measured by our LC-MS/MS method (in our hand) and by Medical Care Proteomics Biotechnology Co.,Ltd. (company for clinical laboratory testing). The correlation coefficient was determined from the values obtained by two methodologies.
(TIF)

S8 Fig Detection of N- and C-terminal fragments of FLAG-FPN1-myc protein cleaved by NS3-4A protease. Cells with or without HCV infection were transfected with the FLAG-FPN1-myc expression plasmid together with the NS3-4A expression plasmid or the empty vector (EV). At 2 dpt, cell lysates were subjected to western blotting using the antibodies against FPN1, FLAG, c-myc, HCV Core, HA and GAPDH. MG132 and NH_4_Cl were added at final concentrations of 10 μM and 10 mM, respectively, 12 h before cell harvesting.
(TIF)

S9 Fig Amino acid sequence alignment of human and mouse FPN1 around the cleavage site mediated by HCV NS3-4A. Residues of mouse FPN1 identical to human FPN1 are indicated by dots. Arrow: cleavage site by HCV NS3-4A. Underlined: consensus residues at the cleavage site by NS3-4A.
(TIF)

S10 Fig Intracellular iron concentration in FPN1-expressing cells with or without HCV infection. At 1 dpi, Huh7.5.1 cells with or without HCV infection were transfected with the plasmid expressing FPN1 or an empty vector (EV). At 2 dpt, intracellular iron concentrations were measured using the Nitroso-PSAP method. Values obtained from EV-transfected cells without HCV infection were set as 100%.
(TIF)

S11 Fig Confirmation of HCV Core and NS3 expression in the livers of mice introducing recombinant adenoviruses expressing HCV proteins or GFP. FPN1 including its cleaved form (red arrow), HCV Core and NS3 were detected in homogenized liver tissues from four mice expressing GFP (lanes 1–4), Core-NS2 (lanes 5–8), NS3-4A (lanes 9–12) and both Core-NS2 and NS3-4A (lanes 13–16), as described in the legend for Fig 5C–5E, by western blotting.
(TIF)

S12 Fig Induction of hepcidin and BMP6 mRNA expression in the livers of HCV protein-expressing mice. Hepcidin and BMP6 mRNAs in livers of mice injected with recombinant AdVs, as described in the legend for Fig 5C and 5E, were determined by qRT-PCR. Results represent the means with SD from four independent mouse samples. Student’s *t* test; *P<0.05, **P<0.01.
(TIF)

S13 Fig Iron store levels in mouse liver tissues expressing HCV proteins. Three groups of mice (N = 4 each) were created in which recombinant adenoviruses were injected as follows: GFP group as a negative control; AdEFLNLGFP, AdEFNCre and AdEFGFP, Core-NS2 group; AdEFLNLHCVCore/NS2 and AdEFNCre, Core-NS2 and NS3-4A group; AdEFLNLHCVCore/NS2, AdEFNCre, and AdEFHCVNS3/4A. (A) Thin section slices were prepared from livers at 5 dpi and stained with Berlin blue. Representative images of two mouse livers from each group are shown. (B) Total iron scores of 10 fields of view in the livers were individually evaluated by means of histological hepatic iron index. Results represent the plots separately for each group. Welch’s *t* test; *P<0.05, **P<0.01. (C) Hepcidin and BMP6 mRNAs in the livers were determined by qRT-PCR. Results represent the means with SD from four independent mouse samples. Student’s t test; **P<0.01. (D) FPN1 including its cleaved form (red arrow), HCV Core, and NS3 in homogenized liver tissues from four mice expressing both HCV Core-NS2 and HCV NS3-4A were detected by western blotting.
(TIF)

S14 Fig Iron and hepcidin-25 concentrations in sera of mice expressing HCV Core-NS2 or GFP. Serum samples were prepared from C57/BL6J mice injected with AdV expressing HCV Core-NS2 or GFP into the tail vein as described Fig 2G. (A) Serum hepcidin-25 concentrations were measured by Medical Care Proteomics Biotechnology Co.,Ltd. (B) Serum iron concentrations were measured by Oriental Yeast Co., Ltd.
(TIF)

S15 Fig Expression of HCV NS3-4A from genotypes 1–3 does not affect hepcidin and FPN1 mRNA expression. Cells were transfected with a plasmid expressing the HA HCV NS3-4A derived from the viral genotype1b, 2a or 3a or the empty vector (EV), respectively. At 2 dpt, the mRNAs of hepcidin, FPN1, GAPDH were measured using qRT-PCR. HA-tagged NS3-4A, and GAPDH were analyzed by western blotting. Results represent the means with SD from three independent measurements. Student’s *t* test; ns. P>0.05.
(TIF)

S16 Fig Knockdown of hepcidin and FPN1 does not affect HCV replication. After 1 day of HCV infection, siRNA targeted to hepcidin (Silencer Select siRNA, s33739), FPN1 (Silencer Select siRNA, s26902) or a universal negative control siRNA (sicont) at 10 nM was introduced into Huh7.5.1 cells. At 2 dpt, (A) the expression of hepcidin mRNA and FPN1 mRNA and (B) HCV RNA copies were analyzed using qRT-PCR. Results represent the means with SD from three independent measurements. Student’s *t* test; ***P <0.001, ns. P>0.05.
(TIF)

S1 Table Demographic and clinical characteristics of the hepatitis C patients for whom serum hepcidin-25 was measured. The duration of each regimen for DAA treatment was as follows. SOF/LDV: Sofosbuvir/Ledipasvir for 12 weeks. OMV/PTV/r: Ombitasvir/Paritaprevir/Ritonavir for 12 weeks. EBR+GZR: Elbasvir + Grazoprevir for 12weeks. GLE/PIB: Glecaprevir/Pibrentasvir for 8 weeks. Post-treatment blood tests were performed 12 weeks after the end of treatment (SVR12) ND: not detectable.
(TIF)

S2 Table Oligonucleotide sequences used for RT-PCR and ChIP assays. (TIF)

S3 Table List of antibodies used in this study. (TIF)
