# Supplementary material for: Role of hepcidin upregulation and proteolytic cleavage of ferroportin 1 in hepatitis C virus-induced iron accumulation
Source: PLoS Pathog. 2023 Aug 16;19(8):e1011591. doi: 10.1371/journal.ppat.1011591 (PMC10461841; doi:10.1371/journal.ppat.1011591)
Supplement: S1 Text — (DOCX) [file ppat.1011591.s001.docx]

Role of hepcidin upregulation and proteolytic cleavage of ferroportin 1 in hepatitis C virus-induced iron accumulation

Authors

Kazuyoshi Ohta, Masahiko Ito, Takeshi Chida, Kenji Nakashima, Satoshi Sakai, Yumi Kanegae, Hideya Kawasaki, Takuya Aoshima, Shuji Takabayashi, Hirotaka Takahashi, Kazuhito Kawata, Ikuo Shoji, Tatsuya Sawasaki, Takafumi Suda, Tetsuro Suzuki

S1 Text

**Materials and Methods**

**Reagents**

Recombinant human BMP6 was purchased from PeproTech, Inc. (Cranbury, NJ, USA).

**Plasmids**

To make an expression plasmid for HCV Core-NS2 C113S which contains a substitution mutation in the active center of the NS2 protease, the GENEART Site-Directed Mutagenesis System (Thermo Fisher Scientific) and the Dpn I method (New England Biolabs, Ipswich, MA, USA) was used. pcDNA-BMP6, which expresses full-length BMP6, was constructed by inserting PCR products of corresponding BMP6 regions, which were amplified with Huh7.5.1-derived cDNA as a template, into pcDNA3.1. To make a plasmid expressing FLAG-tagged HCV Core, a sense primer containing the FLAG tag at the N-terminus of Core was used for PCR with pCAG-Core as a template. To create pcDNA-FLAG-FPN1-myc, a PCR product obtained with an antisense primer containing the c-myc tag at the C-terminus of FPN1 and pcDNA-FLAG-FPN1 as a template was inserted into pcDNA3.1.
