# Supplementary figures and images for: Role of hepcidin upregulation and proteolytic cleavage of ferroportin 1 in hepatitis C virus-induced iron accumulation
Source: PLoS Pathog. 2023 Aug 16;19(8):e1011591. doi: 10.1371/journal.ppat.1011591 (PMC10461841; doi:10.1371/journal.ppat.1011591)

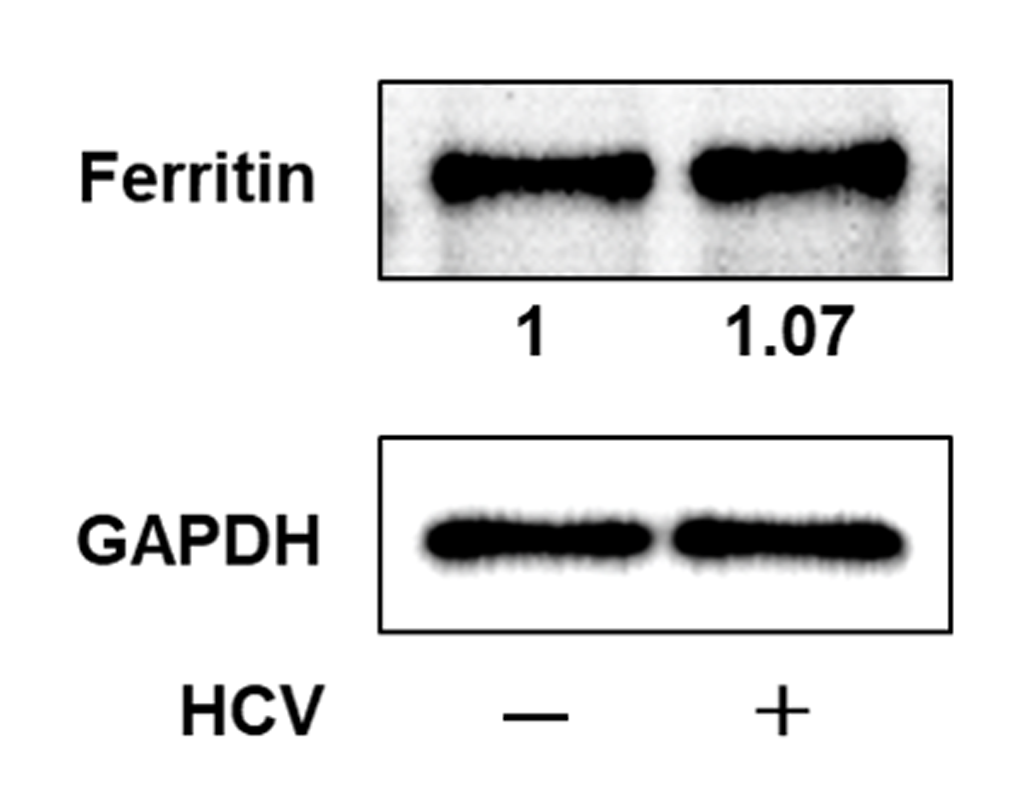

Supplement: S1 Fig — The protein levels of ferritin in cells with or without HCV infection as described Fig 1C were detected by western blotting. Intensities of the protein bands were quantified by ImageJ. (TIF) [file ppat.1011591.s002.tif]

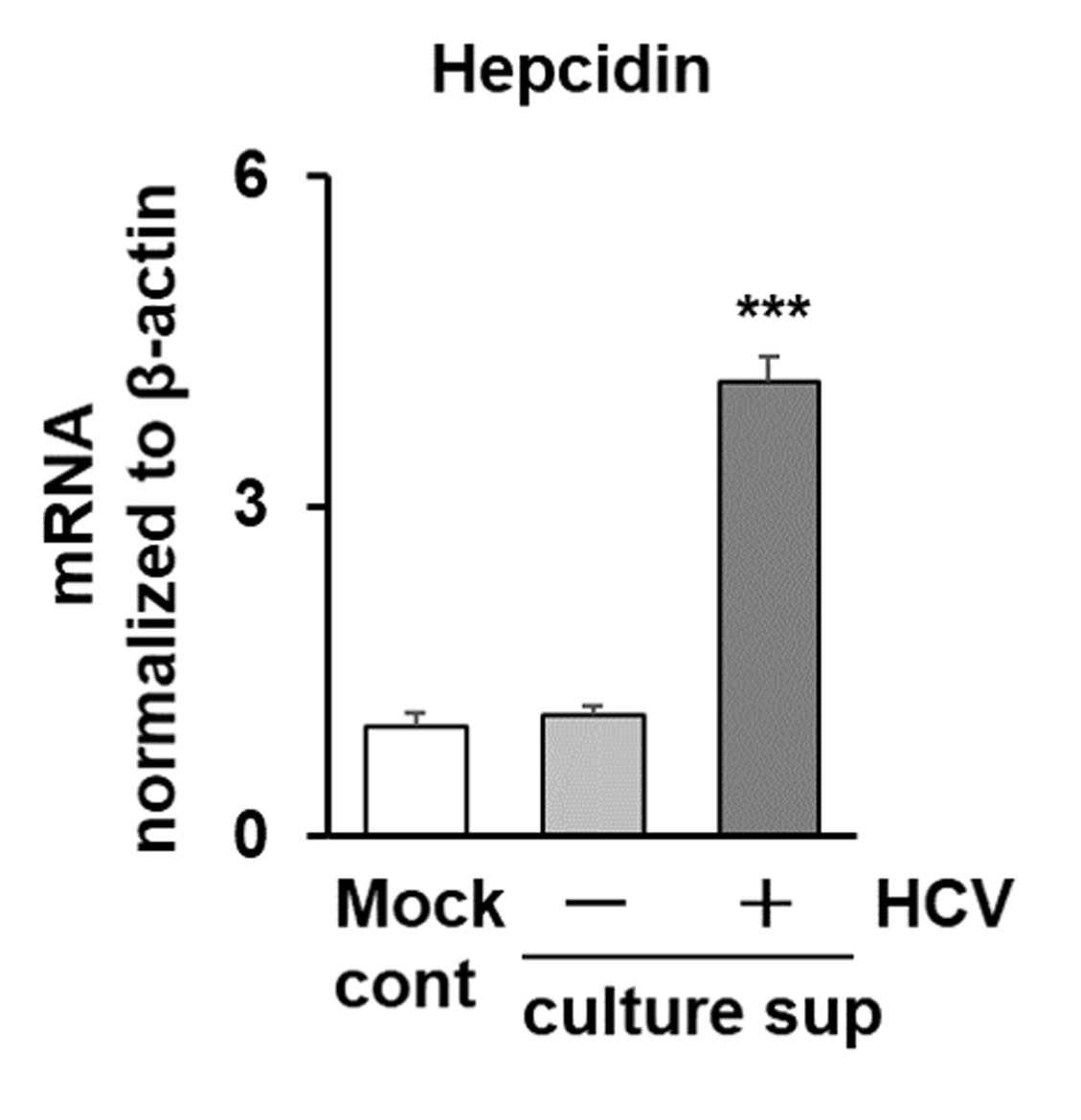

Supplement: S2 Fig — Concentrates of culture supernatants of Huh7.5.1 cells with or without HCV infection were prepared by the same ultrafiltration method used for the preparation of HCV stock. After 3 days of inoculation with the concentrate from HCV-infected cells (+) or uninfected cells (-) to naïve Huh7.5.1 cells, intracellular hepcidin mRNA levels were analyzed using qRT-PCR. Results represent the means with SD from three independent measurements. Student’s t test; ***P <0.001. (TIF) [file ppat.1011591.s003.tif]

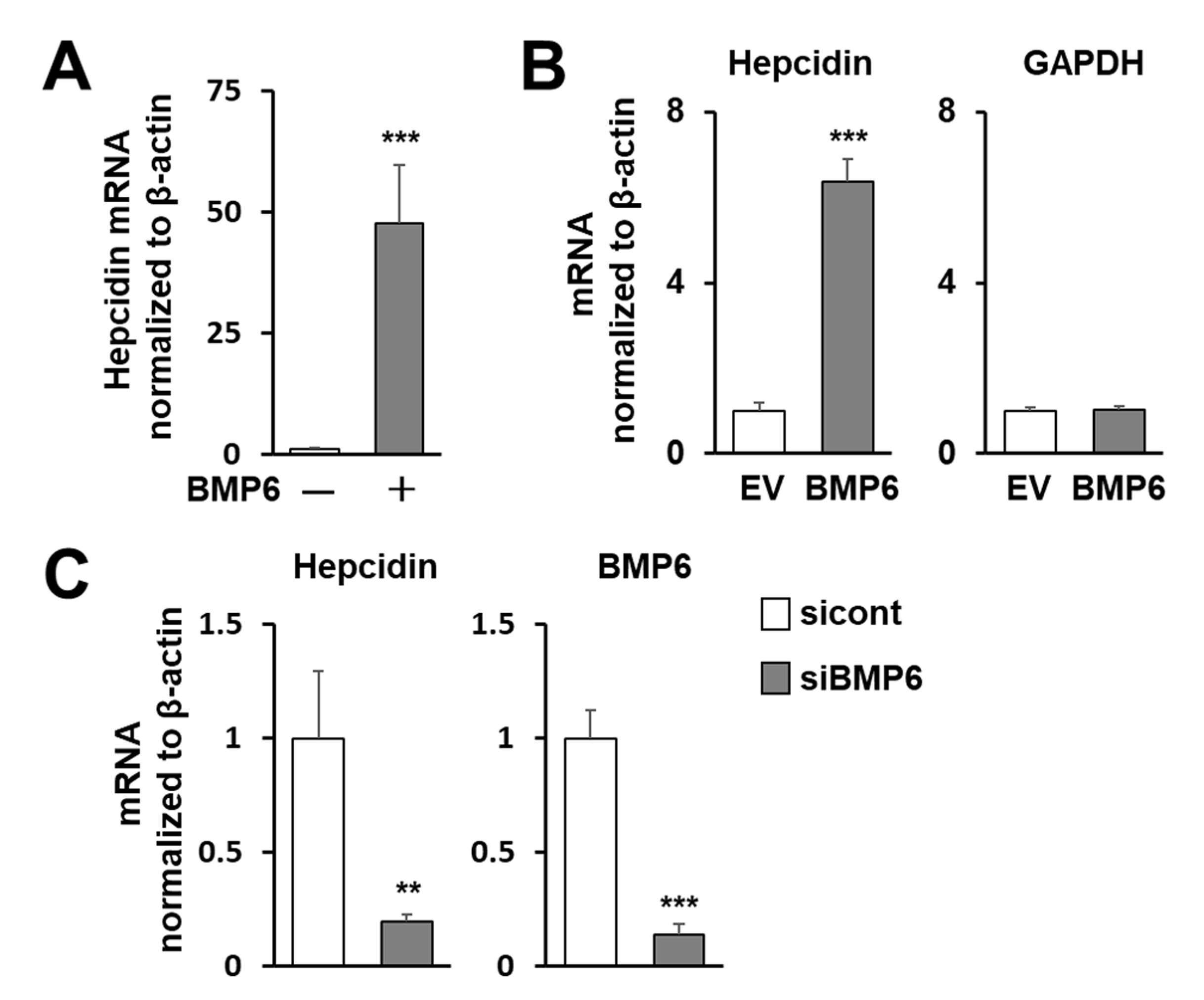

Supplement: S3 Fig — (A) Recombinant BMP6 at a final concentration of 100 ng/mL (+) or vehicle (-) was added to Huh7.5.1 cells. After 48 h, total RNAs were prepared, and hepcidin mRNA was analyzed by qRT-PCR. (B) Cells were transfected with a plasmid expressing BMP6 or an empty vector (EV). At 2 dpt, total RNAs were extracted from cells, and mRNAs of hepcidin and GAPDH were quantified by qRT-PCR. (C) siRNA targeted to BMP6 (Silencer Select siRNA, s2032) or a universal negative control siRNA (sicont) at 10 nM was introduced into Huh7.5.1 cells. At 2 dpt, total RNAs were extracted from cells, and mRNAs of hepcidin and BMP6 were quantified by qRT-PCR. Results represent the means with SD from three independent cultured cell samples. Student’s t test; **P<0.01, ***P<0.001. (TIF) [file ppat.1011591.s004.tif]

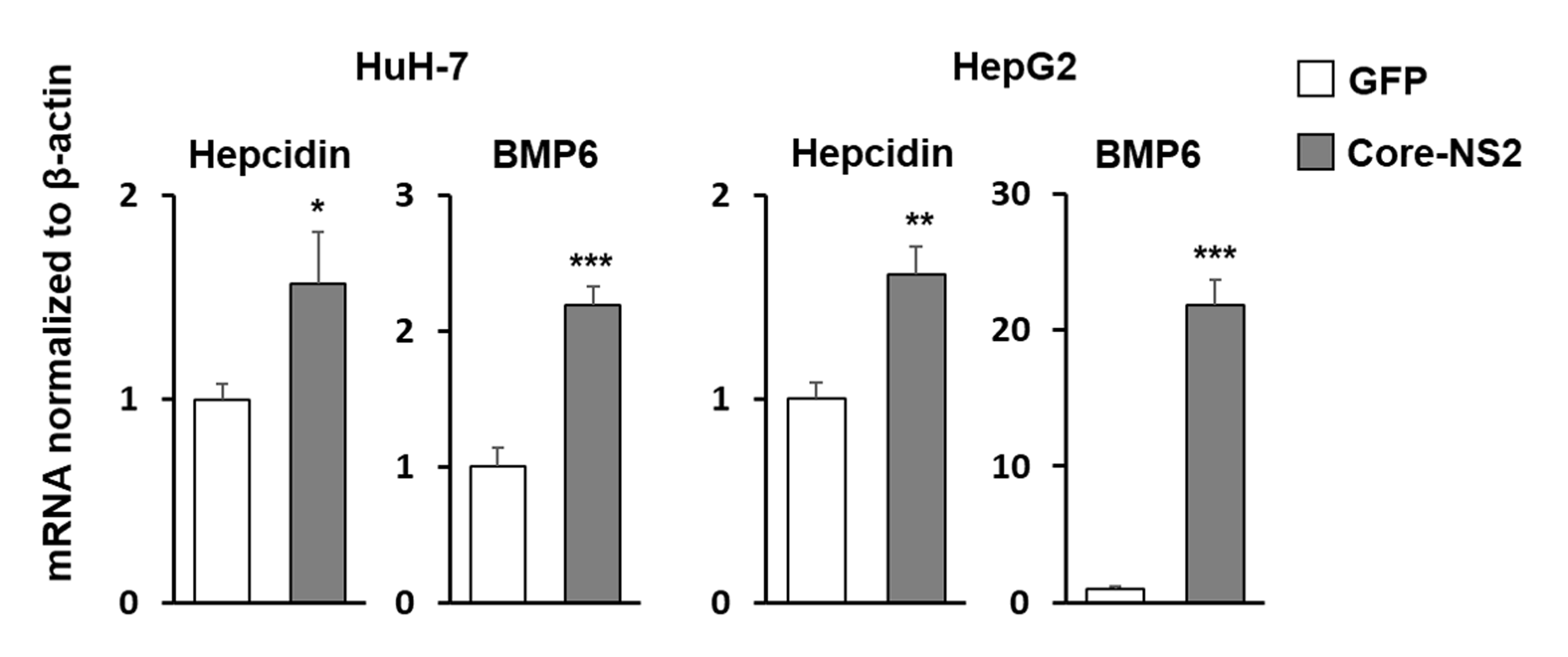

Supplement: S4 Fig — HCV Core-NS2 or GFP-expressing plasmid was transfected into HuH-7 cells or HepG2 cells. At 2 dpt, total RNAs were extracted from cells, and mRNAs of hepcidin and BMP6 were quantified by qRT-PCR. Student’s t test; *P<0.05, **P<0.01, ***P<0.001. (TIF) [file ppat.1011591.s005.tif]

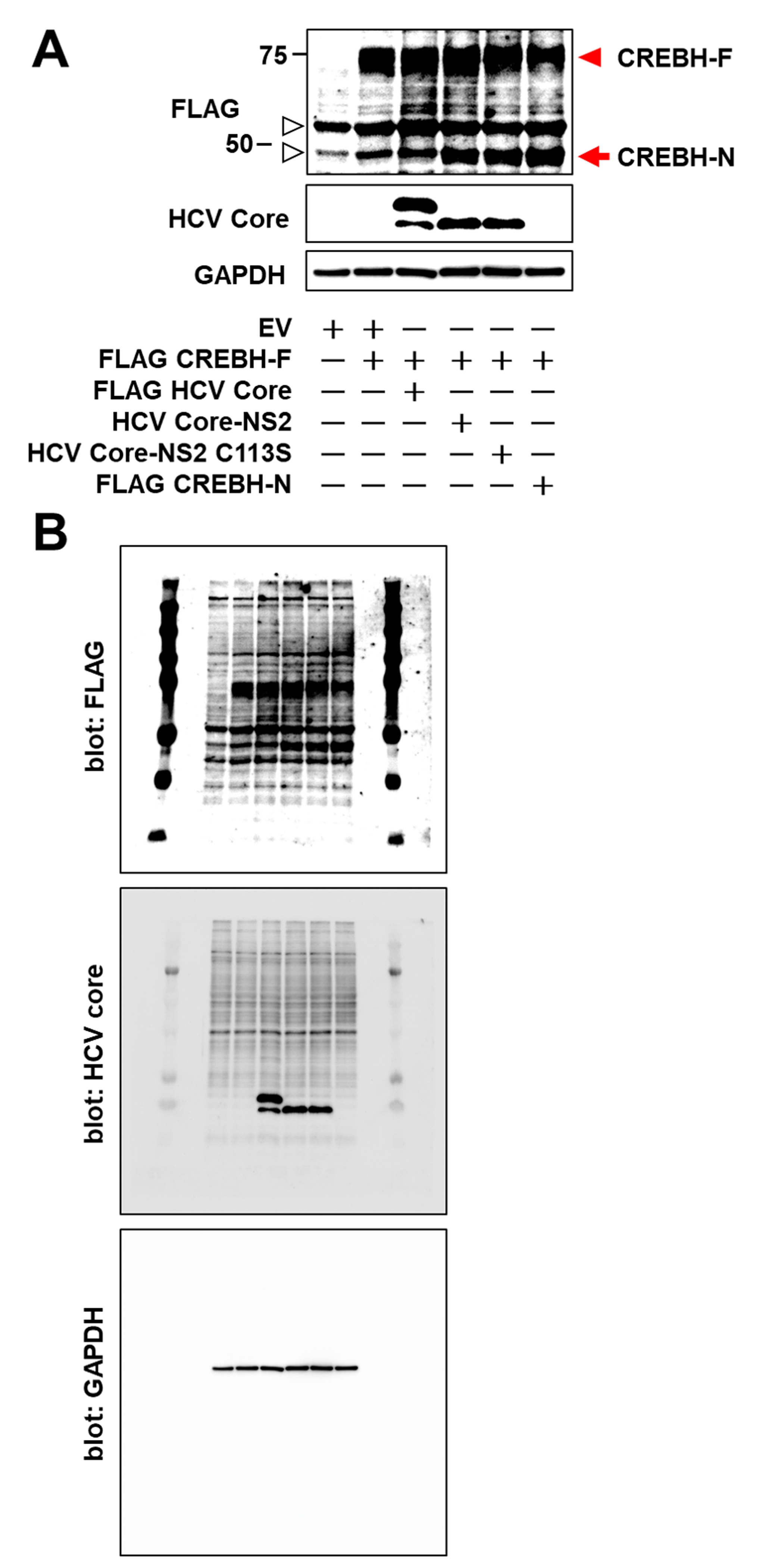

Supplement: S5 Fig — (A) Cells were transfected with a plasmid expressing the full-length FLAG-tagged CREBH (CREBH-F) together with FLAG-tagged HCV Core, HCV Core-NS2 and HCV Core-NS2 C113S expressing vector or the empty vector (EV). At 2 dpt, proteolytically-processed activated CREBH (CREBH-N) was analyzed by western blotting. Cells transfected with the plasmid expressing the FLAG-tagged CREBH-F (red arrowhead) together with FLAG-tagged CREBH-N (red arrow) were used as a control. Open triangles, nonspecific proteins detected. (B) indicates the original data used for (A). (TIF) [file ppat.1011591.s006.tif]

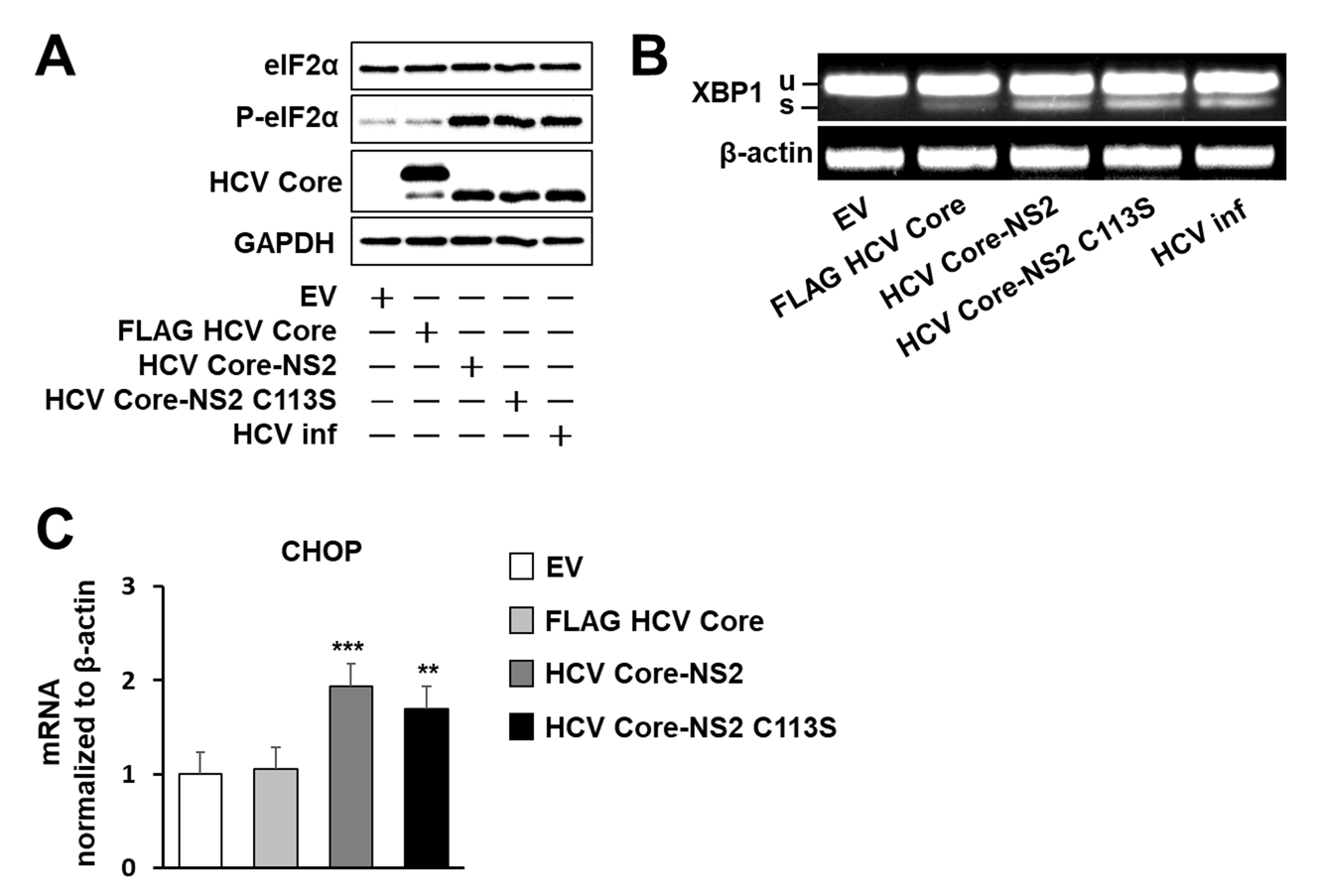

Supplement: S6 Fig — (A) Huh7.5.1 cells were transfected with the FLAG HCV Core, HCV Core-NS2, HCV Core-NS2 C113S expression plasmid or the empty vector (EV). The cells with HCV infection were also prepared. After 3 days of transfection or infection, eukaryotic initiation factor 2α (eIF2α), p-eIF2α, HCV Core and GAPDH were analyzed by western blotting. (B-C) Total RNAs from cells as described in (A) were extracted by the TRI reagent (Molecular Research Center, Cincinnati, OH, USA) and were transcribed using the SuperScript VILO cDNA Synthesis Kit (Thermo Fisher Scientific). (B) The splicing of x-box binding protein 1 (XBP1) mRNA was detected by RT-PCR using specific primers. PCR products were separated by electrophoresis on 2% agarose gels and visualized by ethidium bromide staining. (C) mRNA expression of CCAAT enhancer-binding protein (C/EBP)-homologous Protein (CHOP) was analyzed using qRT-PCR. Results represent the means with SD from three independent measurements. Student’s t test; **P<0.01, ***P <0.001. (TIF) [file ppat.1011591.s007.tif]

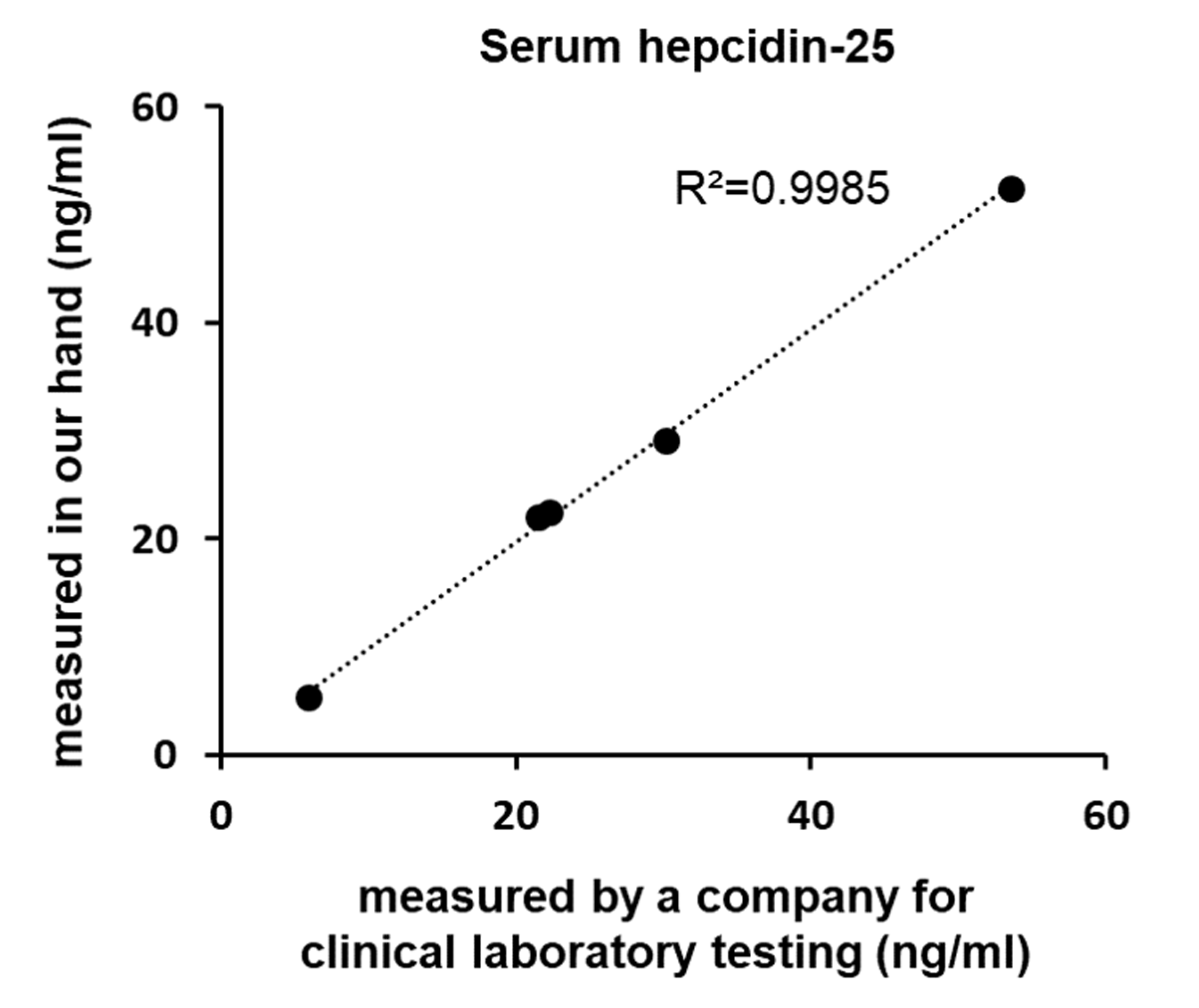

Supplement: S7 Fig — Sera from five hepatitis C patients before DAA therapies were used. Serum hepcidin concentrations were measured by our LC-MS/MS method (in our hand) and by Medical Care Proteomics Biotechnology Co.,Ltd. (company for clinical laboratory testing). The correlation coefficient was determined from the values obtained by two methodologies. (TIF) [file ppat.1011591.s008.tif]

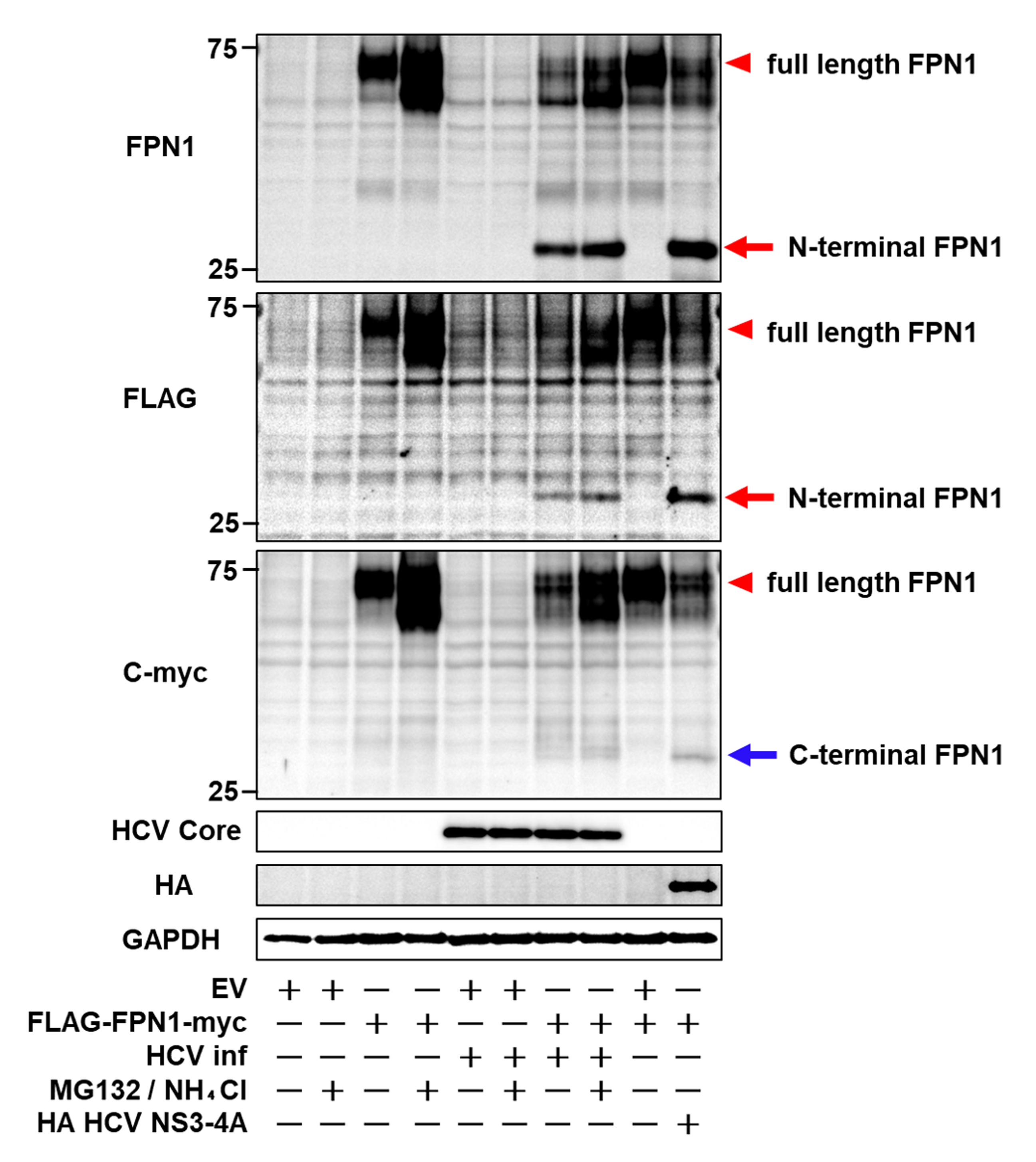

Supplement: S8 Fig — Cells with or without HCV infection were transfected with the FLAG-FPN1-myc expression plasmid together with the NS3-4A expression plasmid or the empty vector (EV). At 2 dpt, cell lysates were subjected to western blotting using the antibodies against FPN1, FLAG, c-myc, HCV Core, HA and GAPDH. MG132 and NH4Cl were added at final concentrations of 10 μM and 10 mM, respectively, 12 h before cell harvesting. (TIF) [file ppat.1011591.s009.tif]

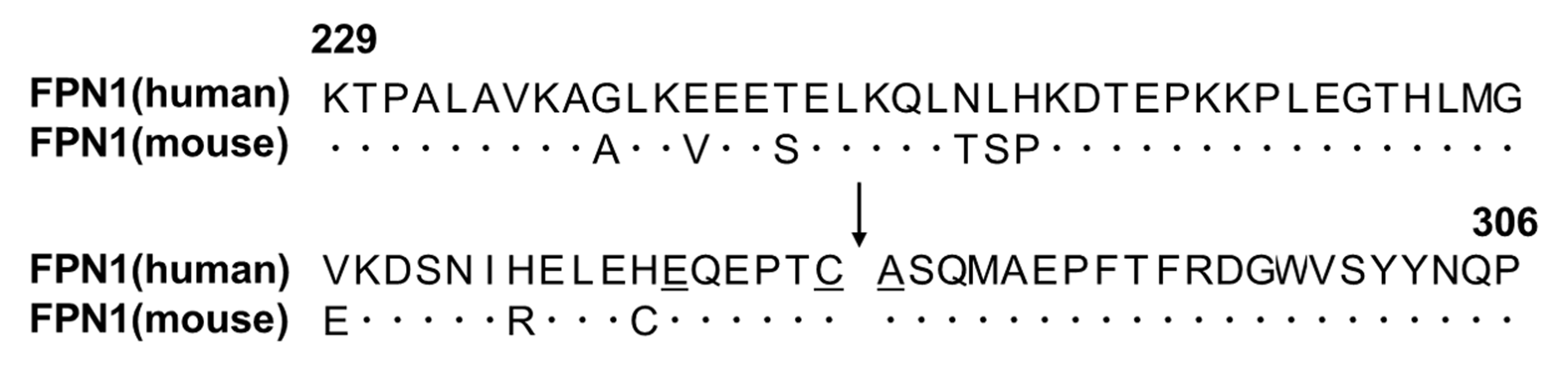

Supplement: S9 Fig — Residues of mouse FPN1 identical to human FPN1 are indicated by dots. Arrow: cleavage site by HCV NS3-4A. Underlined: consensus residues at the cleavage site by NS3-4A. (TIF) [file ppat.1011591.s010.tif]

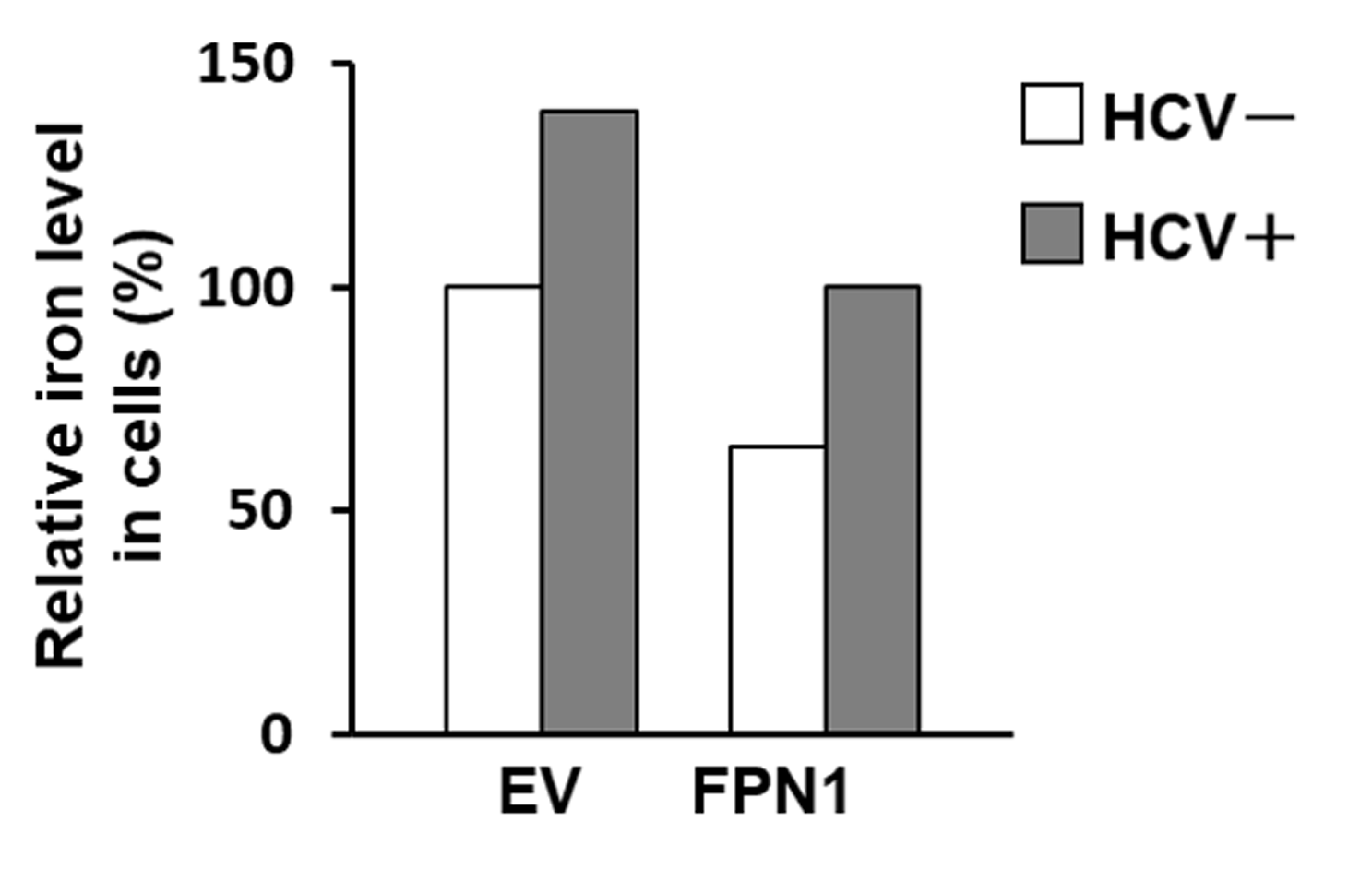

Supplement: S10 Fig — At 1 dpi, Huh7.5.1 cells with or without HCV infection were transfected with the plasmid expressing FPN1 or an empty vector (EV). At 2 dpt, intracellular iron concentrations were measured using the Nitroso-PSAP method. Values obtained from EV-transfected cells without HCV infection were set as 100%. (TIF) [file ppat.1011591.s011.tif]

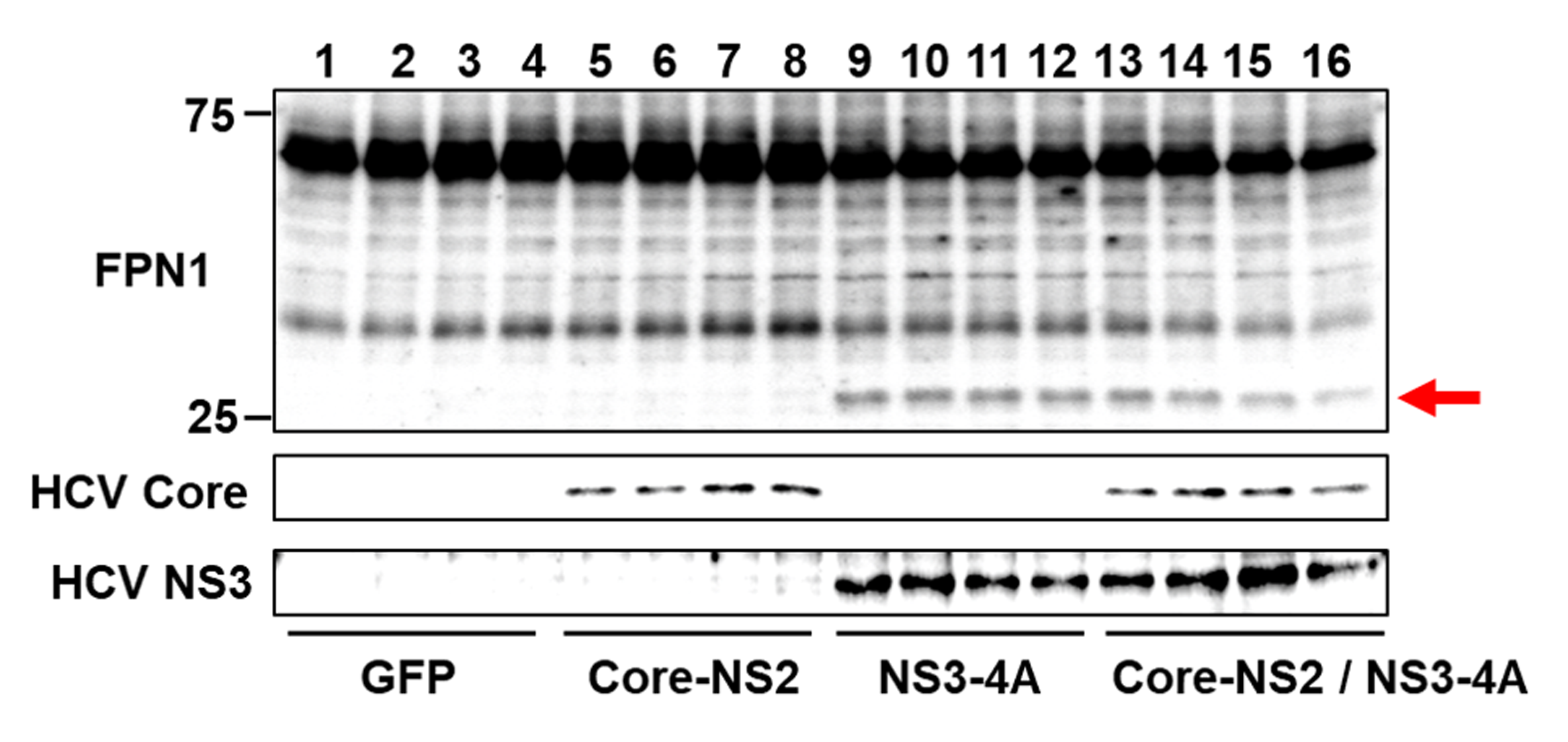

Supplement: S11 Fig — FPN1 including its cleaved form (red arrow), HCV Core and NS3 were detected in homogenized liver tissues from four mice expressing GFP (lanes 1–4), Core-NS2 (lanes 5–8), NS3-4A (lanes 9–12) and both Core-NS2 and NS3-4A (lanes 13–16), as described in the legend for Fig 5C–5E, by western blotting. (TIF) [file ppat.1011591.s012.tif]

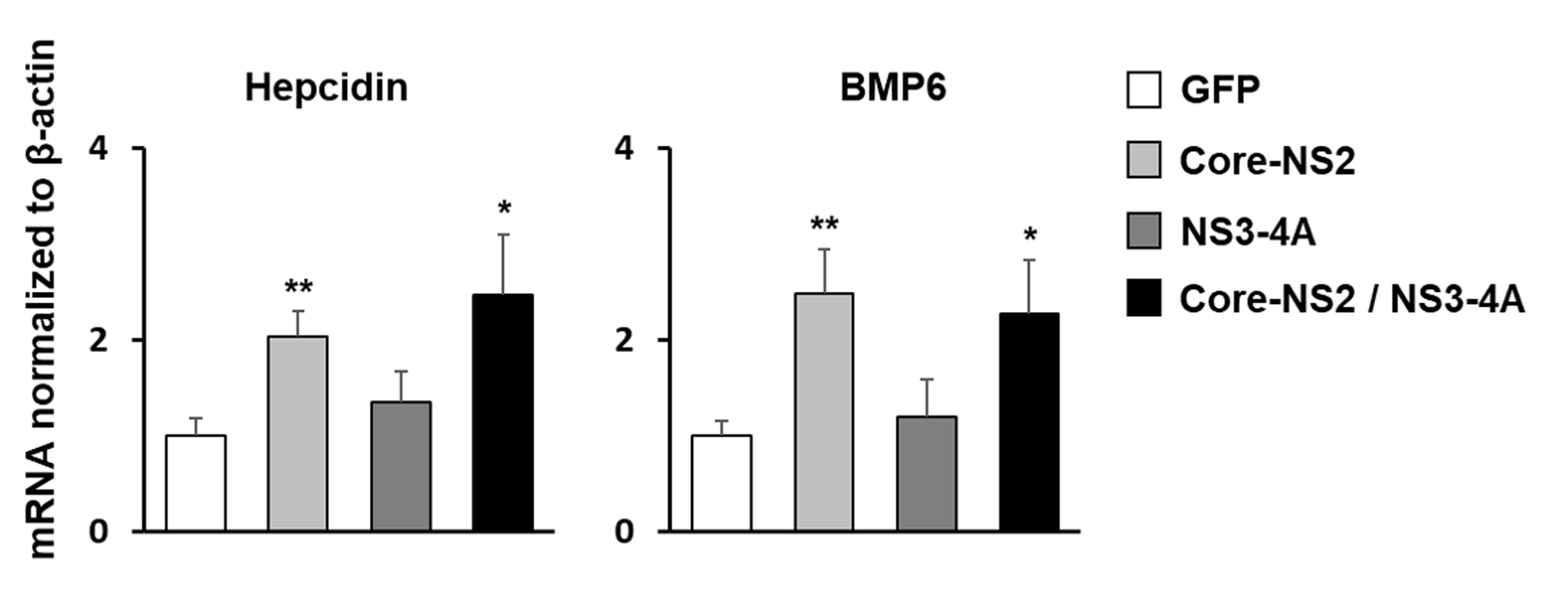

Supplement: S12 Fig — Hepcidin and BMP6 mRNAs in livers of mice injected with recombinant AdVs, as described in the legend for Fig 5C and 5E, were determined by qRT-PCR. Results represent the means with SD from four independent mouse samples. Student’s t test; *P<0.05, **P<0.01. (TIF) [file ppat.1011591.s013.tif]

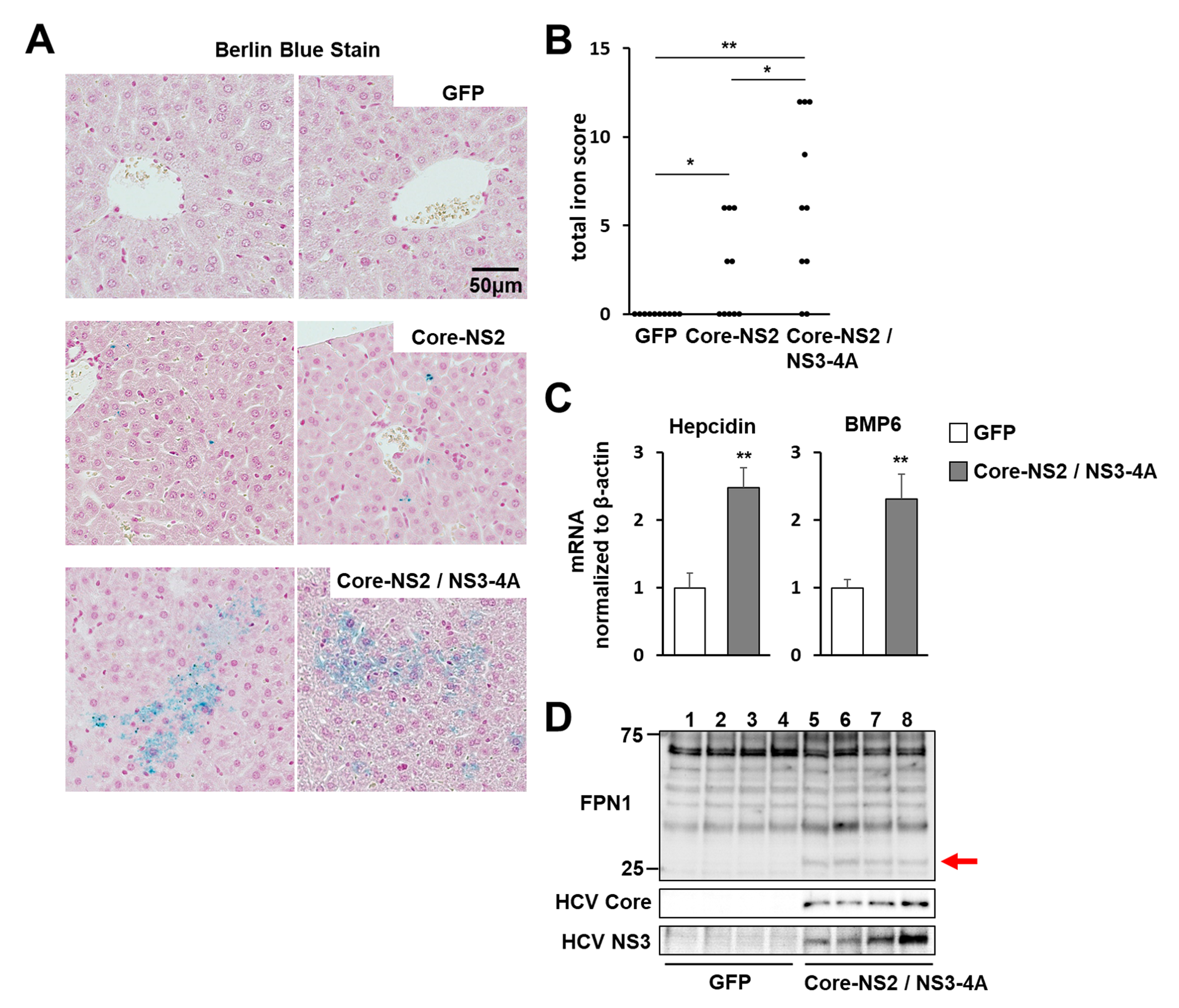

Supplement: S13 Fig — Three groups of mice (N = 4 each) were created in which recombinant adenoviruses were injected as follows: GFP group as a negative control; AdEFLNLGFP, AdEFNCre and AdEFGFP, Core-NS2 group; AdEFLNLHCVCore/NS2 and AdEFNCre, Core-NS2 and NS3-4A group; AdEFLNLHCVCore/NS2, AdEFNCre, and AdEFHCVNS3/4A. (A) Thin section slices were prepared from livers at 5 dpi and stained with Berlin blue. Representative images of two mouse livers from each group are shown. (B) Total iron scores of 10 fields of view in the livers were individually evaluated by means of histological hepatic iron index. Results represent the plots separately for each group. Welch’s t test; *P<0.05, **P<0.01. (C) Hepcidin and BMP6 mRNAs in the livers were determined by qRT-PCR. Results represent the means with SD from four independent mouse samples. Student’s t test; **P<0.01. (D) FPN1 including its cleaved form (red arrow), HCV Core, and NS3 in homogenized liver tissues from four mice expressing both HCV Core-NS2 and HCV NS3-4A were detected by western blotting. (TIF) [file ppat.1011591.s014.tif]

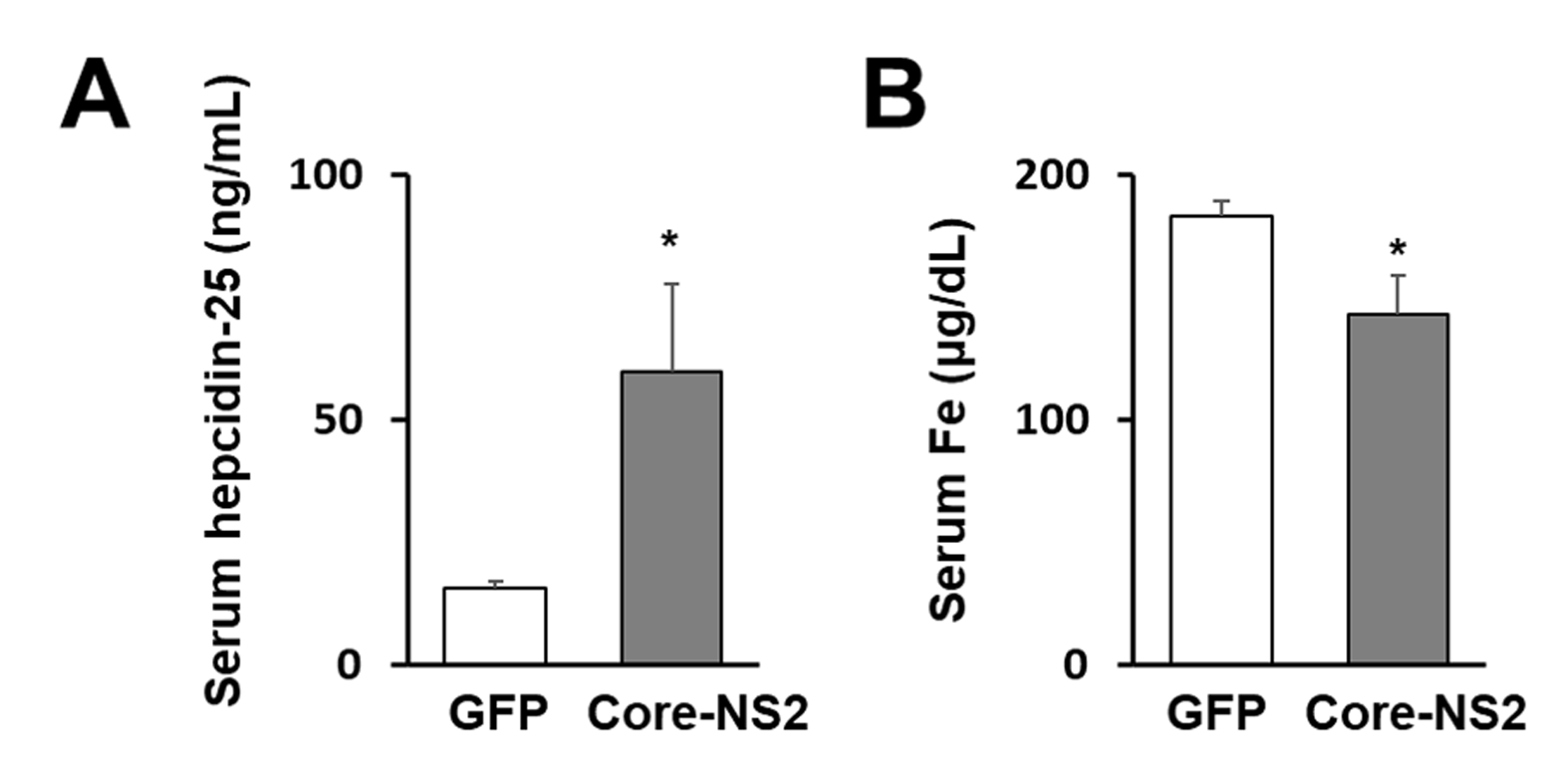

Supplement: S14 Fig — Serum samples were prepared from C57/BL6J mice injected with AdV expressing HCV Core-NS2 or GFP into the tail vein as described Fig 2G. (A) Serum hepcidin-25 concentrations were measured by Medical Care Proteomics Biotechnology Co.,Ltd. (B) Serum iron concentrations were measured by Oriental Yeast Co., Ltd. (TIF) [file ppat.1011591.s015.tif]

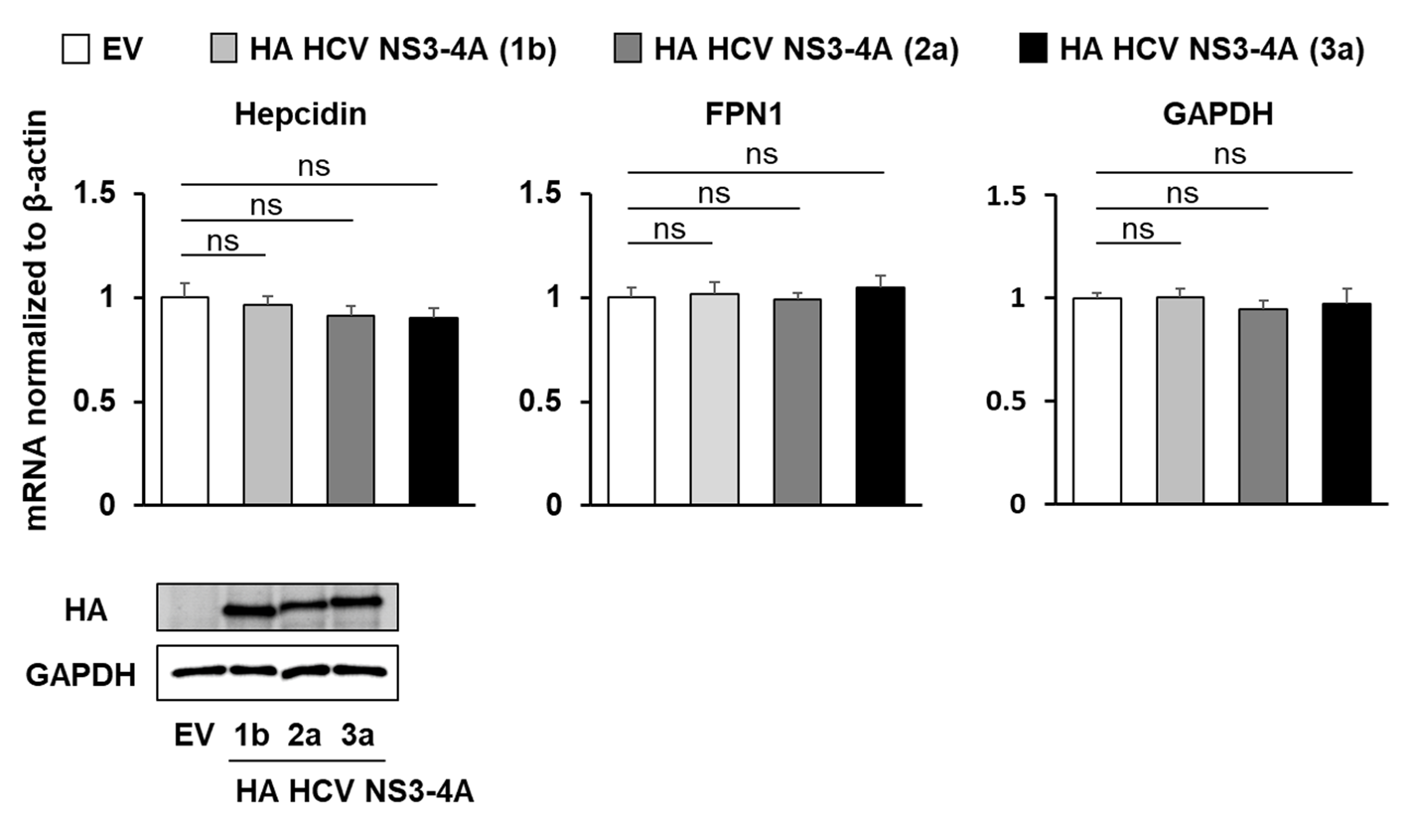

Supplement: S15 Fig — Cells were transfected with a plasmid expressing the HA HCV NS3-4A derived from the viral genotype1b, 2a or 3a or the empty vector (EV), respectively. At 2 dpt, the mRNAs of hepcidin, FPN1, GAPDH were measured using qRT-PCR. HA-tagged NS3-4A, and GAPDH were analyzed by western blotting. Results represent the means with SD from three independent measurements. Student’s t test; ns. P>0.05. (TIF) [file ppat.1011591.s016.tif]

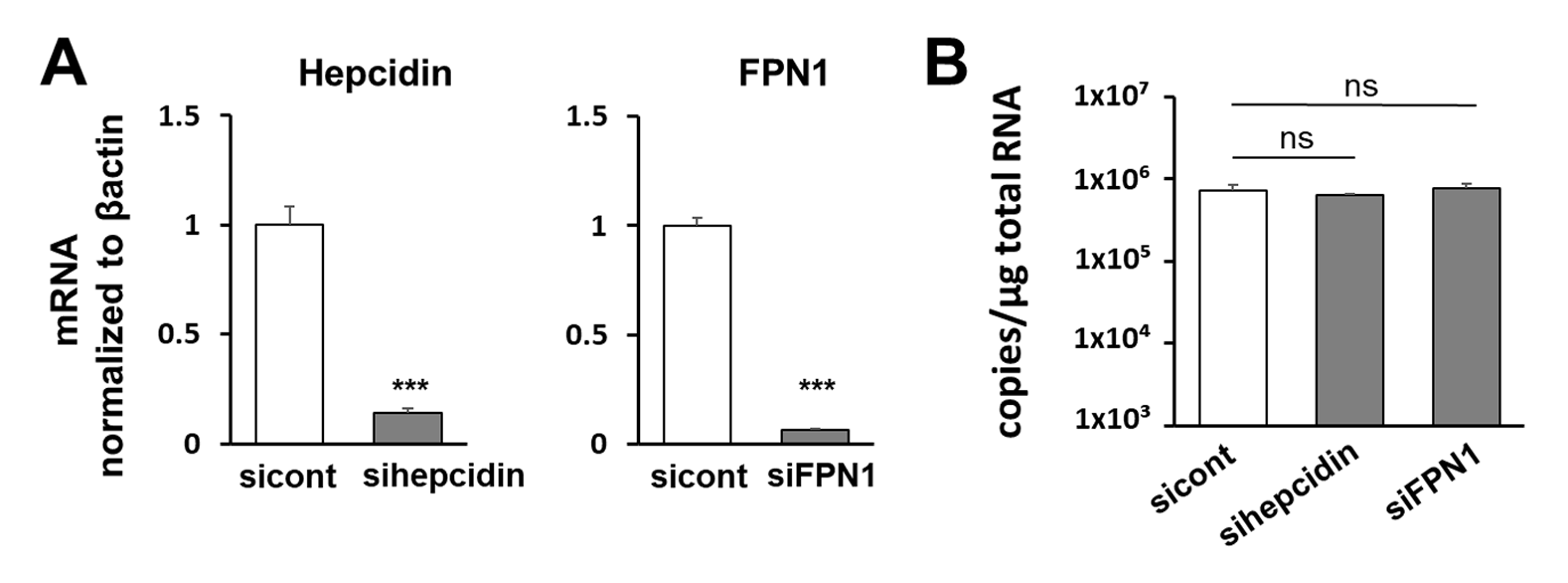

Supplement: S16 Fig — After 1 day of HCV infection, siRNA targeted to hepcidin (Silencer Select siRNA, s33739), FPN1 (Silencer Select siRNA, s26902) or a universal negative control siRNA (sicont) at 10 nM was introduced into Huh7.5.1 cells. At 2 dpt, (A) the expression of hepcidin mRNA and FPN1 mRNA and (B) HCV RNA copies were analyzed using qRT-PCR. Results represent the means with SD from three independent measurements. Student’s t test; ***P <0.001, ns. P>0.05. (TIF) [file ppat.1011591.s017.tif]

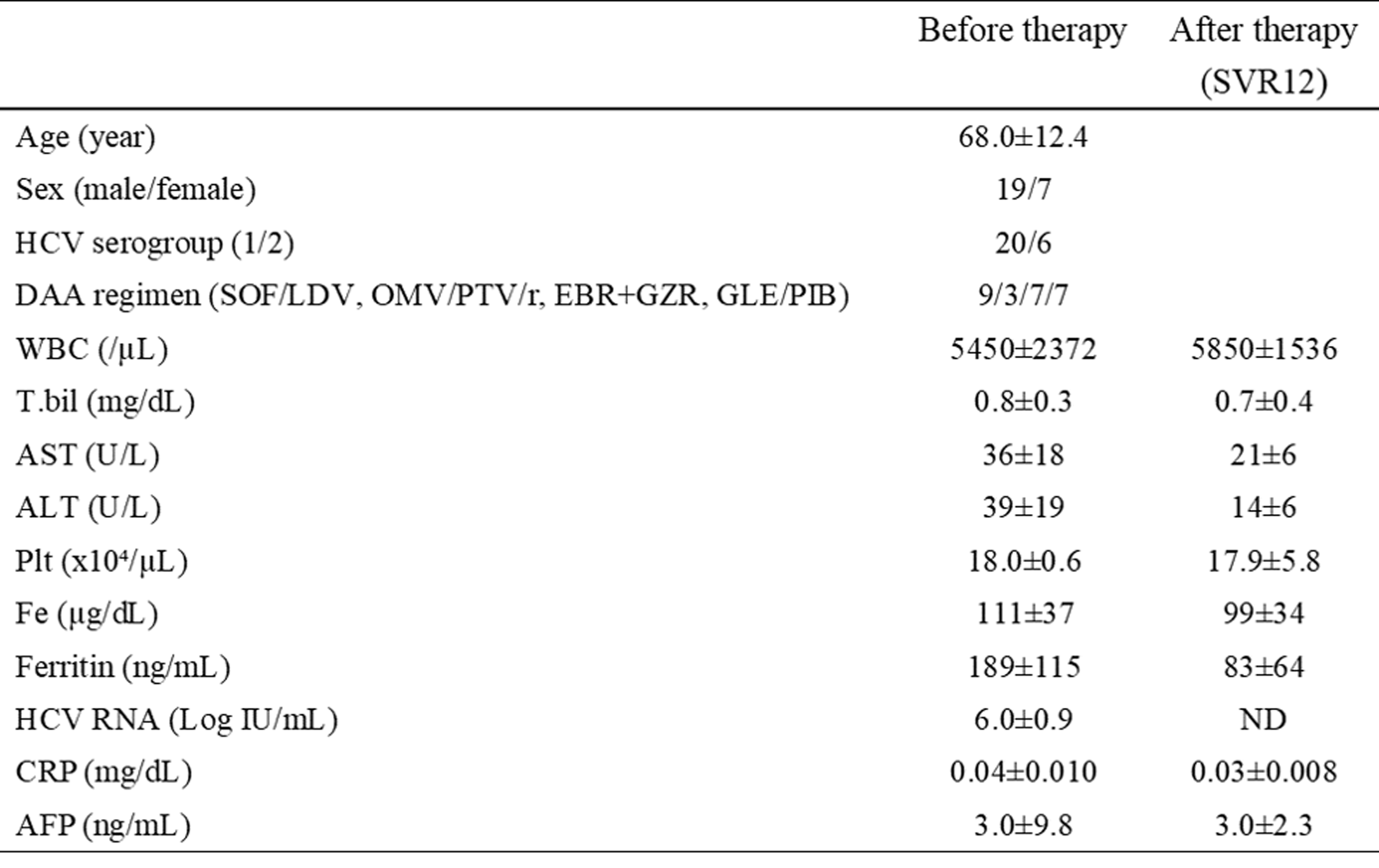

Supplement: S1 Table — The duration of each regimen for DAA treatment was as follows. SOF/LDV: Sofosbuvir/Ledipasvir for 12 weeks. OMV/PTV/r: Ombitasvir/Paritaprevir/Ritonavir for 12 weeks. EBR+GZR: Elbasvir + Grazoprevir for 12weeks. GLE/PIB: Glecaprevir/Pibrentasvir for 8 weeks. Post-treatment blood tests were performed 12 weeks after the end of treatment (SVR12) ND: not detectable. (TIF) [file ppat.1011591.s018.tif]

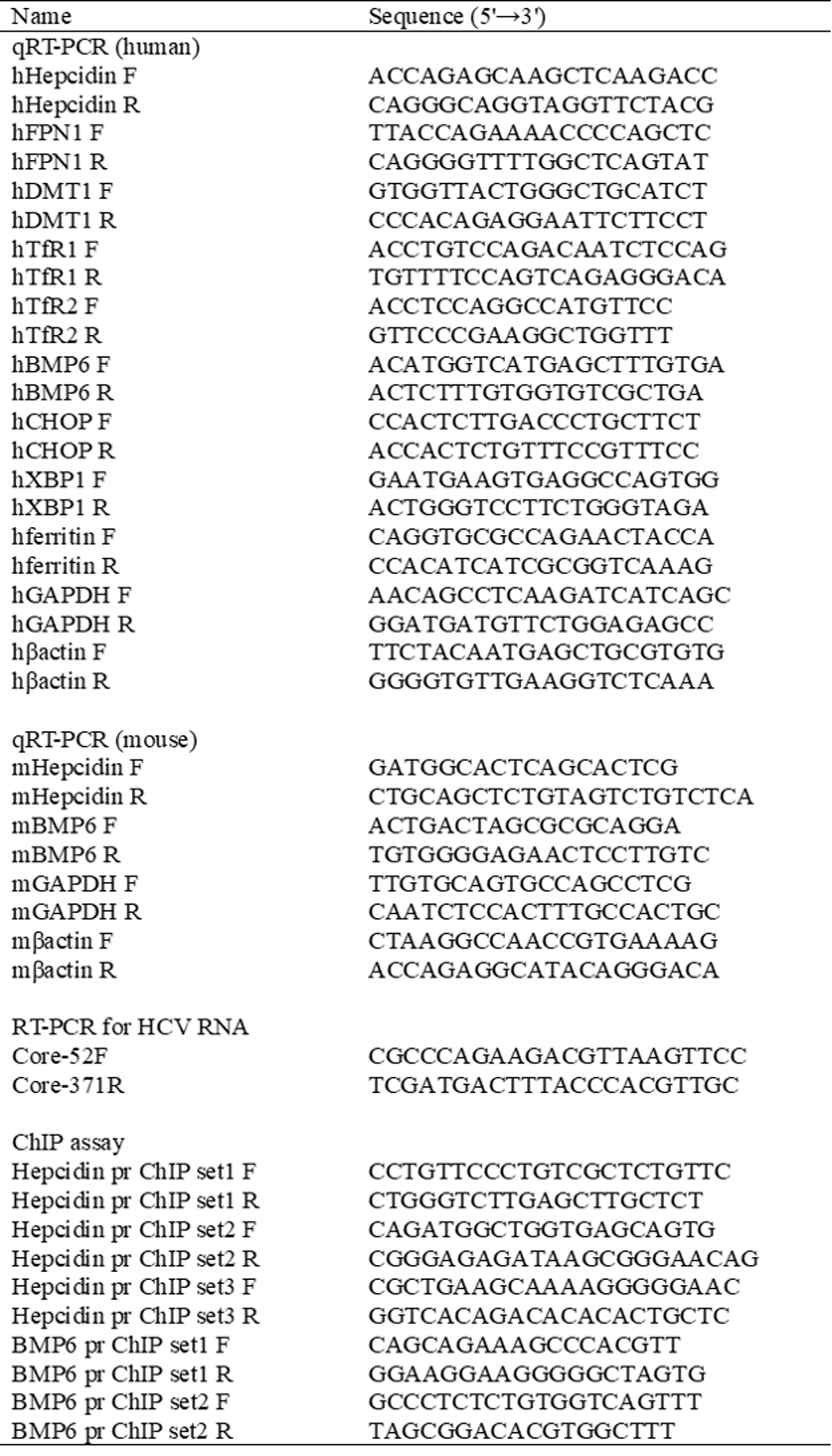

Supplement: S2 Table — (TIF) [file ppat.1011591.s019.tif]

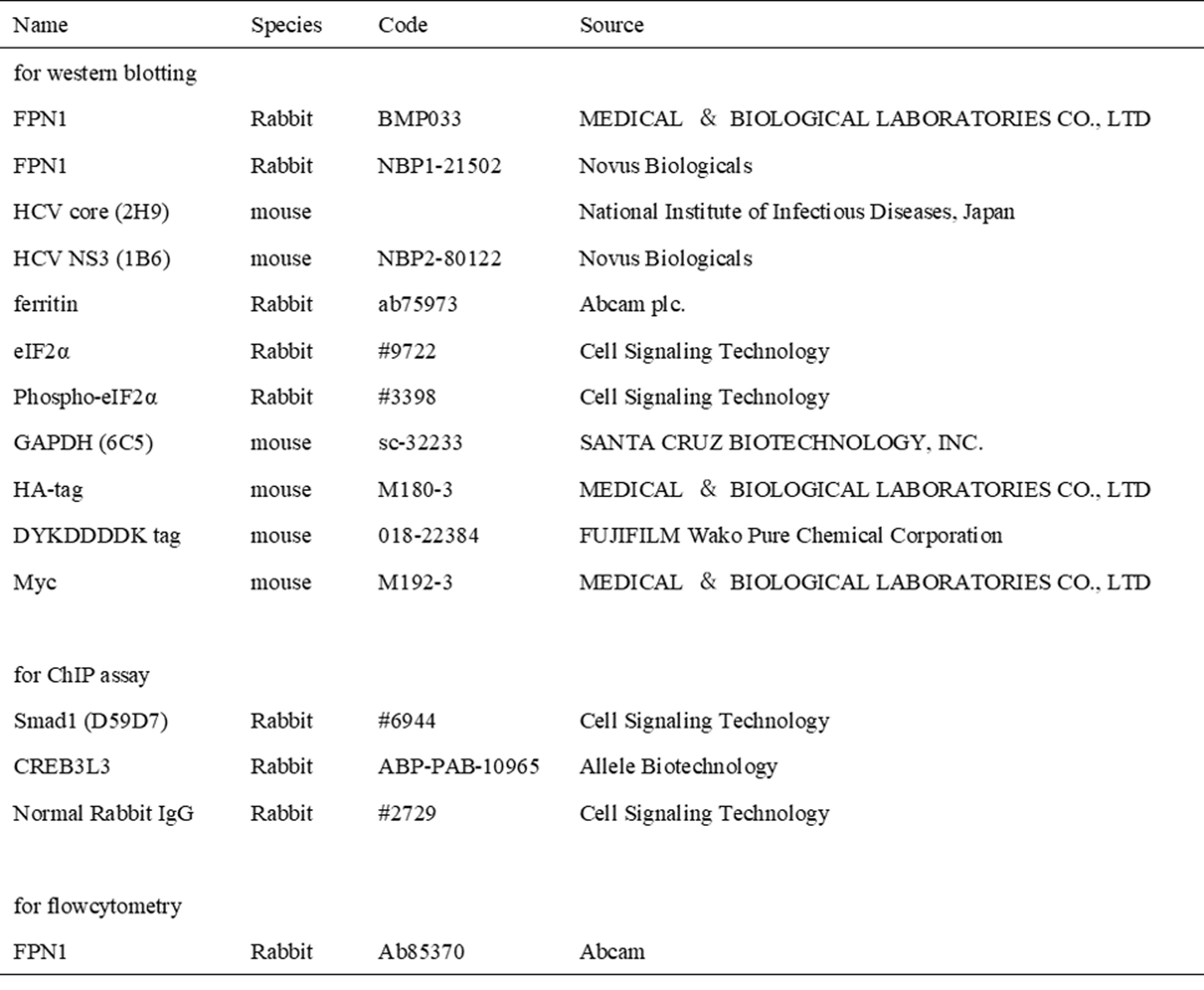

Supplement: S3 Table — (TIF) [file ppat.1011591.s020.tif]
